# *NeuroTransDB*: highly curated and structured transcriptomic metadata for neurodegenerative diseases

**DOI:** 10.1093/database/bav099

**Published:** 2015-10-15

**Authors:** Shweta Bagewadi, Subash Adhikari, Anjani Dhrangadhariya, Afroza Khanam Irin, Christian Ebeling, Aishwarya Alex Namasivayam, Matthew Page, Martin Hofmann-Apitius, Philipp Senger

**Affiliations:** ^1^Department of Bioinformatics, Fraunhofer Institute for Algorithms and Scientific Computing (SCAI), Schloss Birlinghoven, 53754 Sankt Augustin, Germany,; ^2^Rheinische Friedrich-Wilhelms-Universitaet Bonn, Bonn-Aachen International Center for Information Technology, 53113, Bonn, Germany,; ^3^Department of Chemistry, South University of Science and Technology of China, No 1088, Xueyuan Road, Xili, Shenzhen, China,; ^4^Luxembourg Centre for Systems Biomedicine, University of Luxembourg, 7, avenue des Hauts-Fourneaux, L-4362 Esch-sur-Alzette, Luxembourg and; ^5^Translational Bioinformatics, UCB Pharma, 216 Bath Rd, Slough SL1 3WE, United Kingdom

## Abstract

Neurodegenerative diseases are chronic debilitating conditions, characterized by progressive loss of neurons that represent a significant health care burden as the global elderly population continues to grow. Over the past decade, high-throughput technologies such as the Affymetrix GeneChip microarrays have provided new perspectives into the pathomechanisms underlying neurodegeneration. Public transcriptomic data repositories, namely Gene Expression Omnibus and curated ArrayExpress, enable researchers to conduct integrative meta-analysis; increasing the power to detect differentially regulated genes in disease and explore patterns of gene dysregulation across biologically related studies. The reliability of retrospective, large-scale integrative analyses depends on an appropriate combination of related datasets, in turn requiring detailed meta-annotations capturing the experimental setup. In most cases, we observe huge variation in compliance to defined standards for submitted metadata in public databases. Much of the information to complete, or refine meta-annotations are distributed in the associated publications. For example, tissue preparation or comorbidity information is frequently described in an article’s supplementary tables. Several value-added databases have employed additional manual efforts to overcome this limitation. However, none of these databases explicate annotations that distinguish human and animal models in neurodegeneration context. Therefore, adopting a more specific disease focus, in combination with dedicated disease ontologies, will better empower the selection of comparable studies with refined annotations to address the research question at hand. In this article, we describe the detailed development of *NeuroTransDB, *a manually curated database containing metadata annotations for neurodegenerative studies. The database contains more than 20 dimensions of metadata annotations within 31 mouse, 5 rat and 45 human studies, defined in collaboration with domain disease experts. We elucidate the step-by-step guidelines used to critically prioritize studies from public archives and their metadata curation and discuss the key challenges encountered. Curated metadata for Alzheimer’s disease gene expression studies are available for download.

**Database URL:** www.scai.fraunhofer.de/NeuroTransDB.html

## Background

Considerable effort by the global research community has been dedicated to addressing a limited understanding of the pathogenic events underlying neurodegenerative disease (NDD) (1, 2). The cumulative output of these efforts has established an increased amount of deposited molecular data and published knowledge. As life expectancy continues to rise and treatment options for NDD remain limited, there is an increasing urgency to translate this amassed molecular data into biomarker tools for early diagnosis; to open the possibility of disease altering and preventative therapy (3, 4). Furthermore, biomarkers aiding the decision-making process for therapies targeting specific pathophysiological mechanisms will help to address the high drug attrition rate in the NDD pharmaceutical industry. Informatic efforts to facilitate the integration and interrogation of the distributed molecular data legacy for NDD can enable a systematic and objective prioritization of molecular protagonists and therefore potential biomarkers in NDD (5–8).

In this direction, we have previously developed a semantic framework, called *NeuroRDF* (9), for integration of heterogeneous molecular data types, extracted from biomedical literature, transcriptomic repositories and bespoke databases. *NeuroRDF* enables researchers to formulate biological questions that relate to the interplay of different facets of molecular biology as a formalized query. Even today, the most abundant source of quantitative molecular data remains transcriptomic data, which can support hypothesis-free, elucidation of biological function (10). When the same biological function is replicated in additional expression data sets, it increases the plausibility of the derived hypothesis (11).

The inaccessibility of the brain is a significant barrier to molecular analysis of NDD and this frequently limits the availability of samples from post-mortem tissue (12, 13). This is evident when simply comparing the availability of NDD studies to other disease domains, like cancer (14), in public archives such as Gene Expression Omnibus (GEO) (15) and ArrayExpress (16) (see Supplementary Figure S1). For instance, GEO contains 157 NDD studies in contrast to 16,910 cancer studies. Therefore, animal models are an important complement to human-derived samples but are at best an incomplete reflection of the human conditions. Assessing the biological complementarity of studies is important when considering a meta-analysis. Such an assessment can be a cumbersome process as searching in these public repositories is principally based on free text. Additionally, limited adoption of controlled vocabularies, such as the Experimental Factor Ontology (EFO) (17), to describe the metadata fields and lack of compliance to defined standards (18) has contributed to the dilemma. This has resulted in metadata being scattered as unstructured prose in public databases and as additional annotations, widely distributed in originating publications. Moreover, applying automated methods to retrieve information from these databases could compromise on the accuracy. On the other hand, capturing missing annotations through the manual curation can incur huge costs of trained labour.

Capturing the associated metadata in a standardized and precise fashion will empower integrative analysis by helping to control sources of variability that do not relate to the hypothesis under investigation (11, 19–21). Ober *et al.* (22) have reported on differing gene-expression patterns related to gender and suggest gender-specific gene architectures that underlay pathological phenotypes. Li *et al. *(23) observed distinct expression patterns, strongly correlated with tissue pH of the studied subjects; these patterns are not random but dependent on the cause of death: brief or prolonged agonal states. Thus, studies enriched with metadata annotations provide the power to obtain more precise differential estimates.

### Related work

Numerous approaches have been proposed to tackle the problem of identifying relevant gene-expression studies and annotating metadata information resulting in several databases, web servers and data exploration tools. These (value added) databases differ from one another based on their objectives, information content and mode of query.

AnnotCompute (24) is an information discovery platform that allows effective querying and grouping of similar experiments from ArrayExpress, based on conceptual dissimilarity. The dissimilarity measure used, Jaccard distance, which is derived from the MAGE-TAB fields submitted by the data owners. Another tool, Microarray Retriever (MaRe) (25) enables simultaneous querying and batch retrieval from both GEO and ArrayExpress for a range of common attributes (e.g. authors, species) (MAGE-TAB is a submission template, tab-delimited, for loading functional genomics data into ArrayExpress. https://www.ebi.ac.uk/fgpt/magetab/help/). GEOmetadb (26) is a downloadable database of structured GEO metadata with programmatic querying libraries in both R and MATLAB. However, all the above-mentioned resources suffer from a common limitation: they rely completely on the submitted data and do not provide solutions for missing metadata information.

Several value-added databases invest manual curation effort to enrich metadata information for gene-expression studies. Many Microbe Microarrays Database (M^3^D) (27) contains manually curated metadata, retrieved from the originating publications, for three microbial species, conducted on Affymetrix platforms. Similarly, the Oncomine database (28) contains extensive, standardized and curated human cancer microarray data. A-MADMAN (19); an open source web application, mediates batch retrieval and reannotation of Affymetrix experiments contained in GEO for integrative analyses. Microarray meta-analysis database (M^2^DB) (11) contains curated single-channel human Affymetrix experiments (from GEO, ArrayExpress and literature); categorized into five clinical characteristics, representing disease state and sample origin. However, experiments with missing link to the published paper in GEO and ArrayExpress were excluded. A substantial paucity of sample associated gender information in GEO and ArrayExpress motivated Buckberry *et al.* (29) to develop a *R *package, *massiR *(MicroArray Sample Sex Identifier) to label the missing and mislabelled samples retrospectively with gender information, based on data from Y chromosome probes. Apart from publicly available resources, there are various commercial products that contain manually curated transcriptomic metadata: NextBio, Geneve stigator and InSilicoDB (30) (http://www.nextbio.com/b/nextbioCorp.nb and https://genevestigator.com/gv/). However, none of the above databases are optimized to capture detailed metadata specific to neurodegenerative disease. In addition, these databases fail to handle species-specific annotations; especially treatments applied on animal models to partially explicate or treat human-related NDD mechanisms, which may strongly contribute to increase the predictive power of translating preclinical results in NDD drug trials.

Here, we describe the detailed development of *NeuroTransDB, *a manually curated database containing metadata annotations for neurodegenerative studies and an enabling resource for supporting integrative studies across human, mouse and rat species. The participation of our group, at Fraunhofer Institute SCAI, in projects funded by the Neuroallianz Consortium (a part of the BioPharma initiative of the German Ministry of Education and Research) and the evident lack of a comprehensive NDD specific metadata archive has motivated us to develop **Neuro**degenerative **Trans**criptomic **D**ata**B**ase (*NeuroTransDB*) (http://www.neuroallianz.de/en/mission.html)*. *This database now contains more than 20 dimensions of metadata annotations for human studies, as well as mouse and rat models, defined in agreement with disease experts. To demonstrate our approach, we chose to highlight Alzheimer’s disease for this publication because it depicts a wide spectrum of the possible annotations across different types of metadata in neurodegeneration. Additionally, we have applied the same approach to all publicly available Parkinson’s and Epilepsy studies, which shows that the overall approach is unspecific to the disease. However, the curated data for these two diseases will be released in the future under the terms of a Neuroallianz agreement. The database is updated every six months using highly trained curators. An interactive graphical user interface to access this data is currently being developed as part of the AETIONOMY IMI project (http://www.aetionomy.eu).

## Curation of gene-expression studies: prerequisites, key issues and solutions

**Figure 1.**
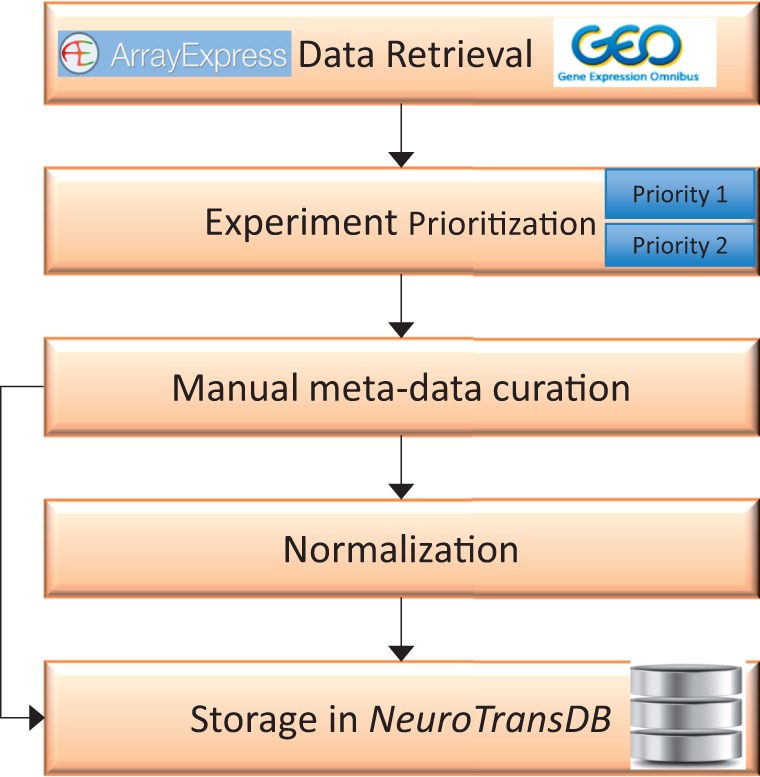
**Overall workflow for curation of gene expression studies related to neurodegeneration from public archives.** The first step involves automated retrieval of gene expression studies (along with metadata) from public archives such as GEO, and ArrayExpress. The related studies were further assigned to one of the two prioritization classes (priority 1 or priority 2), based on the specific experimental variables. Next, manual curation was applied to capture missing metadata information on priority 1 studies. All the harvested metadata was normalized using standard vocabularies. Both raw and normalized data are stored in *NeuroTransDB*.

This section discusses the workflow we followed to retrieve relevant gene-expression datasets and to generate detailed metadata annotations for each study (Figure 1). First, we retrieved all functional genomics studies from GEO and ArrayExpress that reference Alzheimer’s disease (AD) or a set of AD synonyms, along with the provided metadata (*cf.* Data Retrieval section). Each study was then prioritised (*cf.* Experiment Prioritization section) based on the disease relevancy, experimental type and sample source. Only studies in the top prioritization category were subjected to rigorous, semiautomated metadata curation (*cf*. Metadata Curation section). Annotations are standardized by reference to controlled vocabularies for each extracted metadata dimension (*cf.* Normalization of Metadata Annotations section). The curated Alzheimer’s data is stored in *NeuroTransDB*, but in principle the proposed workflow can be applied with little adaptation to any disease indication, especially NDD.

### Primary data resources

Together the GEO and ArrayExpress databases constitute a wealth of gene expression studies and are commonly reused for validating new hypotheses and identifying novel signatures through meta-analysis by multi-data integration (11). GEO is the largest public repository of functional genomic data; maintained by the National Center for Biotechnology Information (NCBI) in the USA. ArrayExpress is the European counterpart of GEO and consists of manually curated experimental information imported from GEO, in addition to the data that are directly submitted by the researchers. To support reuse of the deposited studies, each repository adheres to annotation standards for submission of transcriptomic data: ‘Minimum Information about a Microarray Experiment’ (MIAME) and ‘Minimum Information about a high-throughput nucleotide SEQuencing Experiment’ (MINSEQE) (http://fged.org/projects/miame/ and http://www.fged.org/projects/minseqe/). GEO allows data submission in Excel, SOFT or MINiML format and ArrayExpress as MAGE-TAB through Annotare webform tool (http://www.ncbi.nlm.nih.gov/geo/info/submission.html and http://www.ebi.ac.uk/arrayexpress/submit/overview.html).

### Curation team

An obvious prerequisite for any curation process is to have access to specially trained personnel, who understand the key attributes required to adequately describe an expression experiment and are able to complete these attributes by reference to appropriate resources (31). Such individuals are known as biocurators. We assembled a team of candidate biocurators who have adequate biological experience. Each biocurator underwent extensive training in the fundamentals of curation, including the basics of gene expression study design, outlined by experts, scientists and disease experts. Clear curation guidelines (see Experiment Prioritization and Metadata Curation section) and a weekly meeting of the biocurators with one of the experts ensured good quality, consistency, and uniformity in curation procedure. In addition, this provided an opportunity to get feedback from the biocurators for improving and updating the defined guidelines. To keep abreast and eliminate any bias, the curated data was regularly exchanged between them for good interannotator agreement. The experts resolve any disagreement that may arise between the curators.

### Data retrieval

**Figure 2.**
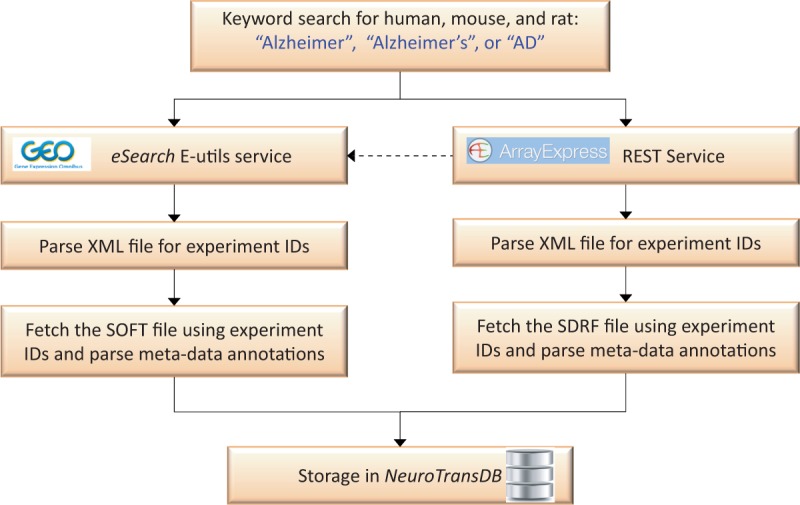
**Automated data retrieval of Alzheimer’s Disease specific gene expression studies from ArrayExpress and GEO.** Here, the dotted line represents the sequence of query performed. Alzheimer’s disease specific experiment IDs were automatically retrieved from GEO and ArrayExpress, using keywords, through *eSearch *and REST service respectively. Metadata information was extracted by automatically parsing sample information files (SDRF and SOFT) of these experiment IDs.

Putative AD studies were programmatically retrieved from GEO and ArrayExpress by applying a recall-optimized keyword search approach, *cf.* Figure 2. The keywords include a set of AD synonyms such as ‘Alzheimer’, ‘Alzheimer’s’ or ‘AD’ in combination with a species filter. Since ArrayExpress imports and curates the majority of GEO experiments, we firstly queried the former through its REST service (http://www.ebi.ac.uk/arrayexpress/help/programmatic_access.html). Conjointly, we further queried GEO using the *eSearch* Entrez Programming Utilities (E-utils) service to fetch additional identifiers (IDs), which were not picked up by the previous query (http://www.ncbi.nlm.nih.gov/geo/info/geo_paccess.html). The final list of unified experiment IDs was downloaded (along with their metadata) and stored in *NeuroTransDB*. Metadata information was captured from Sample and Data Relationship Format (SDRF) file of ArrayExpress and SOFT file of GEO (https://www.ebi.ac.uk/fgpt/magetab/help/creating_a_sdrf.html and http://www.ncbi.nlm.nih.gov/geo/info/soft.html). The above-described steps are fully automated; enabling an automatic update procedure we run every 6 months to obtain new published studies.

### Experiment prioritization

**Figure 3.**
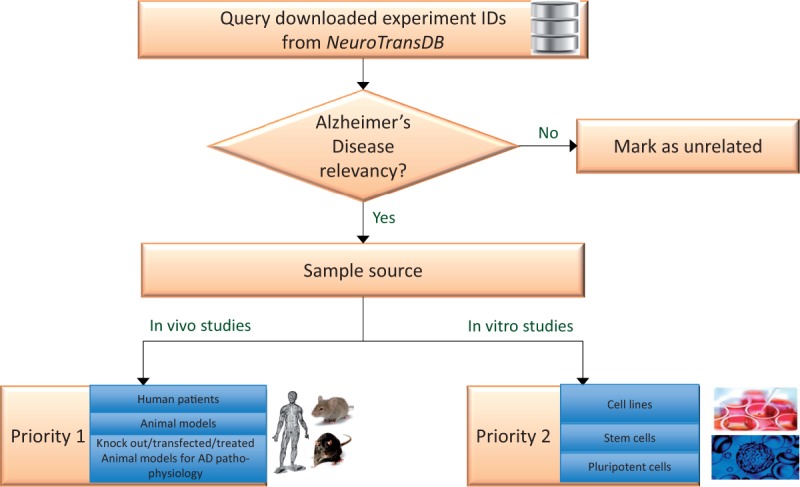
**Experiment prioritization for metadata curation in *NeuroTransDB*.** All the downloaded Alzheimer’s Disease experiments were first checked for their disease relevancy. Those experiments which were falsely retrieved, are marked as unrelated. The remaining experiments were classified into one of two priority classes based on the experiment type: In vivo or In vitro studies. For priority 1, we considered direct/primary samples from human or animal models such as brain tissue, blood, etc. Experiments that were conducted on derived sample sources such as cell lines, were put into priority 2 class.

For integrative meta-analysis, combining studies that address the same objectives could minimize biases from cohort selection (inclusion and exclusion criteria) and other design effects. Anatomical and functional heterogeneity arising from experimental sample source, imposes yet another challenge for integrative analysis. Moreover, keyword-based, recall optimized retrieval of experiments does not guarantee its clinical relevancy to the queried indication or organism. Thus, we propose a straightforward binning approach to select potentially eligible studies for AD as illustrated in Figure 3.

Firstly, we identified experiments relevant to AD indication, if not relevant we mark them as unrelated (referred as AD3 in the database). Relevancy is defined on the basis of the experiment’s characteristics: investigation on AD mechanism, AD associated mechanism, AD genes or contains samples that belong to direct or implicated effects of or on AD. For example, GSE4757 is relevant to AD since it investigates the role of neurofibrillary tangle formation in Alzheimer patients between normal and affected neurons. The retained AD-related experiment IDs were manually classified by biocurators into one of the two- prioritization categories (*cf. *Figure 3). To support this process, a set of classification rules were devised that capture two important considerations: organism specificity and source of the samples used in the study. Although curation with regards to these considerations is of obvious importance, no previously published guidelines were available for reference. To our knowledge, this is the first work where such a guideline has been explicitly detailed. A simplified description of the classification rules adopted for AD disease prioritization is provided below:

Priority 1
  - Experiments that study AD pathophysiology in *in* *vivo* systems
  - Studies containing samples from:
    – Human AD patients such as blood, brain tissue, serum, etc.
    – Animal model samples such as mouse brain tissue or rat brain, e.g. C57BL/6 mice, Sprague–Dawley rat, etc.
    – Animal models modified to study the role of an AD gene (knock-out models), or AD mechanism (transfected models), or diet/drug treatments (treated models), such as TgAPP23, APLP2-KO mice, etc.
  - Experiments containing only healthy/normal samples from human/mouse/rat that are a part of a bigger study investigating AD

Priority 2
  - Experiments that study AD pathophysiology in *in* *vitro* systems
  - Studies containing samples from derived or cultures sources:
    – Cell lines
    – Pluripotent cells
    – Stem cells

#### Incorrect organism or disease specificity

Although the experiment retrieval step was restricted to a specific organism and disease conditions, we observed differing levels of specificity. For example, some mouse studies were retrieved when querying for human studies. Similarly, we obtained experiments for related diseases such as Parkinson’s disease, or diabetes, when querying for AD. Therefore, during study prioritization it was important to confirm the species of origin and relevancy of the study to AD. It’s also possible that keyword-based retrieval may miss AD studies due to incorrect disease or organism tagging. However, we did not perform an exhaustive search for such falsely ignored studies, since it would require immense human effort.

#### Ambiguous species designation

In some studies, human cells such as embryonic stem cells are injected into animal models and post-mortem samples from these animal models are extracted for transcriptomic analysis (e.g. GSE32658 experiment in GEO). Such a study could arguably be classified as either human priority 2 or mouse priority 1. After several discussions, we concluded to prioritize such experiments based on the organism from which the final sample was extracted. In this case, although the mouse was grafted with human tissue, we prioritized it to mouse priority 1.

#### Superseries redundancy

During prioritization, we retrieved several superseries experiments from GEO. Manual inspection revealed that not all the subseries IDs of these superseries experiments were retrieved (see Data Retrieval section) (A SuperSeries is simply a wrapper to group of related Series (typically described in a single publication). It facilitates access to the entire dataset, and establishes a convenient reference entry that can be quoted in the publication (definition provided by the GEO team, as of 27 October 2014) and a subseries is an experiment that is a part of superseries.). With careful manual inspection, we included missing subseries, further subjected to priorization. Conversely, if the inclusion of superseries resulted in the duplication of experiments, we removed the duplicates. Having assigned priority categories to all retrieved AD studies, further metadata curation was focused on the priority 1 studies. Metadata curation steps are described below.

## Metadata curation

Precisely and comprehensively capturing the accessory information for a transcriptomic study as meta-annotations, is an important precursor to identification of comparable experiments that address the biological question at hand. Unfortunately, the current, general, submission standards do not cater to the needs of metadata annotations, specific to a disease domain, during submission. In subsequent sections, we discuss the metadata curation for NDD and key issues faced during the process.

### Metadata annotation fields

We assembled a list of metadata annotations determined to be important for evaluating NDD studies in a process involving consultation by NDD domain experts. All the metadata fields were categorized as organism attributes and sample annotations, based on their relevancy to organism or sample source. Table 1 provides detailed descriptions of curated metadata fields including examples for human, mouse and rat.

**Table 1.** Detailed description of Neurodegenerative disease metadata fields outlined for human, mouse and rat

Annotation type	Metadata fields	Description of the annotation	Relevancy for NDD	Examples	References
Organism attributes	Age	Age of the organism	Main factor for predisposition to disease	84 years, 9 months	(32–35)
	Gender	Gender of the organism	Possible disproportionate effect arising from difference in anatomy and hormonal composition	Male, female	(36, 37)
	Phenotype	Clinical phenotypes of the organism from which the sample was extracted	Supports comparative analysis for underlying pathomechanisms based on the observable/measurable characteristics	Healthy control, early incipient	(38)
	Behavioural Effect	Description of behavioural changes occurring in organism due to treatment or other effects	Impact of developed drug or other environmental factors to treat or reduce the disease/disease symptoms	Reduced agitation/aggression	(39, 40)
	Disease type	The disease occurrence is due to hereditary or effect of environmental factors	To distinguish the genetic variability and complexity between the two types during analysis	Sporadic, familial	(41)
	Stage	Disease stage of the organism from which the sample was extracted	Capability to distinguish severity of the affected disease	Incipient, severe, BRAAK II	(42)
	Cause of death	Reason for the organism’s death	To determine if Alzheimer’s disease or its associated comorbidities are major contributors to death rate	Respiratory disorder	(43)
	Comorbidity	Existence of another disease other than Alzheimer’s	To determine the impact of another disease on Alzheimer’s disease aetiology and progression	Type 2 diabetes	(44, 45)

Sample annotations	Post mortem duration (PMD)	Duration from death till the sample extraction from the dead organism	To assess quality and reliability of the sample obtained by measuring RNA integrity that is influenced by natural degradation of the sample after death	2.5 hours	(46, 47)
	pH	pH value of the extracted post-mortem sample	Indicator of agonal status and RNA integrity	6	(48–50)
	Functional effect	Description of functional effects observed	Observed changes such as gene expression, post-translation, or pathway due to external effects	Decreased expression of BDNF gene, reduced Aβ toxicity	(51, 52)
	Brain region	Brain region of the extracted sample	Provides information of pathogenesis and disease progression, as AD does not affect all the brain regions simultaneously	Hippocampus	(53, 54)
	Cell and cell parts	Type of cells or cell parts extracted from the sample for analysis (if any)	To determine cell type specific expression influencing pathogenesis and regional vulnerability	Synaptoneurosome, neurons and astrocyte	(55, 56)
	Body Fluid	Type of body fluid used for analysis	Could serve as biomarkers for early diagnosis and therapy monitoring	CSF, blood	(57–59)

The table provides a list of metadata fields, confirmed by disease experts, critical for NDD meta-analysis. The selected fields are classified as organism attributes and sample annotations based on their relevancy to organism or sample source.

Several animal models and *in* *vitro* systems have been defined that partially mimic the human diseased conditions. Animal models provide experimentally tractable systems for interrogating NDD, however, not all animal models faithfully mimic human pathophysiology. A dedicated set of meta-annotation was defined for NDD animal models to support assessments of inter-study comparability and translatability to human disease, *cf.* Table 2 These fields were defined with assistance from biologists and disease experts from industry.

**Table 2.** Detailed description of additional metadata fields, defined specifically for mouse and rat models

Annotation type	Metadata fields	Description of the annotation	Relevancy for NDD	Examples	References
Organism attributes	Physical injury	Method used to cause brain injury in animal models	Consideration for analysing plaque formation in animal models to mimic disease symptoms in human	Traumatic brain injury, ischemia reperfusion injury	(60, 61)
	Type of treatment	Description of chemical, drug, genetic or diet treatment	Consideration for determining the effect of treatment on animal models either to mimic or treat the disease/symptoms	Long-term pioglitazone, BDNF treated	(62, 63)
	Dosage	Detailed description of the dosage associated with “type of treatment” description	Consideration of the right quantity of the substance for determining the effect on animal models either to mimic or treat the disease/symptoms	Total polyphenol 6mg/kg/day, received drinking water without ACE inhibitor	(64, 65)
	Mouse/rat strain name	Mouse model official or author given name	To determine the effect of different manipulated animal models in recapitulating key AD features capable of extrapolating to human studies	C57BL/6-129 hybrid, Sprague–Dawley rat	(66, 67)
	Mouse/rat weight	Weight of the animal model during analysis	Establishing a causative link to metabolic disruption	100–150 g	(68)

These additional metadata fields are defined by disease experts as critical for translating mouse/rat model outcomes to human, in the field of neurodegenerative diseases.

### Metadata curation workflow

**Figure 4.**
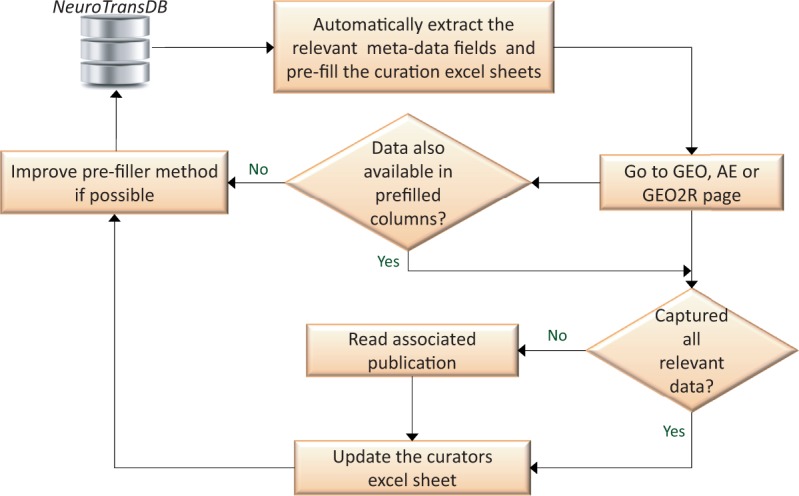
**Semi-automated workflow for metadata curation.** Automatically extracted metadata fields are rechecked by the curators. To capture the missing fields, curators browse through GEO, ArrayExpress (AE) or GEO2R experiment’s description pages. For cases where the information is still incomplete, associated fulltext publications and their associated supplementary material are read. All the extracted metadata annotations are stored in *NeuroTransDB*. Intermediately, if feasible, automated extraction leverages on curator’s experience for improvement. This process is carried out half yearly.

To capture all the relevant meta-annotations, we designed a semiautomated curation workflow, illustrated in Figure 4. Firstly, we automatically retrieved all the available meta-annotations from GEO and ArrayExpress (*cf. *Figure 2). Annotations were captured in an Excel template as shown in Supplementary Figure S2 (A) and confirmed by our trained curators to rectify any inaccuracies.

To capture incomplete and newly defined meta-annotations, we followed a two-step approach. First, we check if the required meta-annotation entries are directly available in GEO, GEO2R or ArrayExpress (http://www.ncbi.nlm.nih.gov/geo/geo2r/). Where the required information is complete, we directly update *NeuroTransDB, *otherwise we move to a second step to manually harvest information for missing annotations. Missing information is retrieved from the originating publications and associated Supplementary files. When necessary, corresponding authors were contacted to request missing entries. The list of experiment IDs where we contacted the authors for further information, along with reason of contact (priority 1 experiments only) are provided in Supplementary Table S1. In most cases, the corresponding author or one of the coauthors responded to our queries; whereas, in few other cases the email addresses no longer remained valid. In the event that the authors do not respond or we were unable to contact them, information in primarily deposited database is used as the final authorative source. Once all the relevant data was captured, we updated the annotations in *NeuroTransDB*. If needed, we updated our automated retrieval iteratively*.*

To demonstrate the metadata curation process, here we relate our experience with study GSE36980 that includes a total of 79 samples. Common MIAME annotations such as gender, age and sample tissue were automatically captured from ArrayExpress and GEO. The associated publication contained further useful information on the enrolled patients, namely: disease stage, post mortem interval before sample extraction and preservation, pathological diagnosis and whether the patient suffered from comorbidities such as diabetes. This information was located in Supplementary File S2 of the associated publication (http://www.ncbi.nlm.nih.gov/pmc/articles/PMC4128 707/ bin/supp_bht101_bht101supp_table2.xls). However, lack of a common ID to enable mapping between the sample entries in GEO and the associated Supplementary File S2 impeded curation. For example, sample GSM907797 in GEO is annotated as being derived from a 95-year-old female patient. However, in their Supplementary file, there are two entries that contain information for patients with same age and gender. The ‘No.’ column, assumed to be patient ID, in the Supplementary file was not helpful for mapping, since it was not mentioned in GEO. Thus, we contacted the authors for the missing link. They provided us an additional Excel sheet where the GEO sample ID was mapped to the ‘No.’ column in the Supplementary file (*cf.* Supplementary Figure S2 (B) and (C)). As a consequence, we achieved a 28.5% increase in the missing metadata information (*cf. *Table 1 for total number of fields) after contacting the authors.

#### Automated meta-annotation retrieval challenges

During automated retrieval of metadata fields, we observed several alternate representations of information for certain annotation types in the archives. For example, age information can be provided in the Characteristics section of GEO or ArrayExpress as ‘age: 57 years’ or ‘Stage IV, male, 57 years’ and so on. We attempted to prenormalize these diverse representations and automatically extract the correct information, however, due to the heterogeneity in data representation, manual curation was still required.

Although ArrayExpress and GEO provide programmatic access to their meta-annotations, much essential information appears in fields meant for general categories. For example, information about the sample source and clinical disease presentation appear in the sample title ‘PBMC mRNA from Alzheimer’s disease patient 2’. Adhering to the standard submission protocol for data entry, this information should appear in the ‘Characteristics column’ of ArrayExpress and GEO. Again inconsistent adherence to annotation standards means that manual inspection is needed to capture correct and complete information from these archives.

#### Accessing linked publications

For annotation information that is not directly available from the source repositories, we refer to the associated full text publications. However, not all deposited studies link to an associated publication in PubMed, contributing to a significant loss of information while curating. We attempted to overcome this by searching for an associated article using the study title with search engines such as SCAIView and/or Google (http://www.scaiview.com and https://www.google.com). Supplementary Figure S3 shows the percentage of articles that were retrieved with different search strategies. We are aware that not all the experiments in these databases are associated with published article (14%), but for 9% of the experiments (prioritized as 1) we were able to link them to publications through a title search. We strongly encourage study depositors to provide PubMed annotation whenever available to allow enhanced meta-annotation. Additionally, database owners should find a more robust way to update their resources.

#### Duplication and inconsistent sample counts

We observed differences in sample counts for some experiments between ArrayExpress and GEO, when downloaded automatically. For example, GSE49160 contained 36 samples in GEO and 72 samples in ArrayExpress. Following closer inspection at several similar experiments, we found that ArrayExpress duplicates sample IDs to provide separate links to different raw file formats or large raw files split into smaller ones (57%), processed raw files (17%), separate entry for each channel in double channel arrays (14%) and replicates (12%) (*cf.* Supplementary Figure S4); moreover, the duplicated samples mostly represented the same annotation information. Since, we used sample IDs as a unique entry in our database, the duplicated IDs were replaced with the last entry from the archive, in* NeuroTransDB,* as read by our algorithm; thus a risk of loosing the raw file or other non-duplicated annotation information.

Apart from duplication, occasionally some samples were missing in one archive relative to the other. For example, GSE47038 had some additional samples in ArrayExpress, which were not present in GEO. When we contacted the ArrayExpress team, they suggested that the experiment entry could be out of sync, since each entry from GEO is uploaded into ArrayExpress only once and is not updated if GEO deletes some samples later. However, they have now corrected the entry. This demonstrates a need for periodic review of study records in each database.

#### Missing RAW filenames

Public transcriptomic archives provide a gateway for the search and retrieval of studies for subsequent analysis outside of the platform. Therefore, one has to obtain the link between a sample’s raw file name and corresponding phenotype. However, this is not the case when applying automated downloads. The majority of the raw file names present in public archives contain syntactical errors such as surrounded by brackets or separated by comma; moreover, such entries could be normalized through a simple script. In cases where no information about sample’s raw file name is provided, manual intervention is required to link sample’s raw file to its respective sample. This clearly indicates the need for standardization of the database entries for automation and to prevent loss of information.

#### Incorrect and incomplete metadata information

We also observed inconsistent meta-annotations between a study deposited in an archive and the information in the linked publication. In GEO for experiment GSE2880, the sample description page states that male Wistar rats have been used for the study. However, when we looked into the associated full text article, in the Methods section, the authors clearly mention using female Wistar rats (69). We are still waiting for the author’s reply to correct the gender information for this entry. Another example is GSE18838, we observe that the ratio of male to female patients provided in GEO (male/female: 19/9) is different from that reported in the Supplementary file (male/female: 18/10); additionally, Supplementary Table S2 provides detailed challenges faced during mapping of age and gender information to samples. When searched in ArrayExpress, this experiment has been removed from the database, for unknown reasons. In yet another example, GSE36980, the age information for sample GSM907823 and GSM907823 vary between GEO (84 and 81 years, respectively) and ArrayExpress (74 and 86 years, respectively). From these anecdotal experiences, it is evident that one has to spend immense effort to obtain correct metadata information. Database owners and the submitters have to take utmost care to provide the correct data for reproducibility.

#### Information extraction from chained references

One further time consuming task included looking following chains of references to previous publications for human and animal model information such as mouse name, cross breeding steps applied and human subject information. In some cases, we had to tediously trace back 5–6 cross-referred publications to obtain the original source of information.

### Normalization of metadata annotations

Meta-annotation involved the curation team extracting the original text as provided in GEO/ArrayExpress or in the published literature. We observed many different ways to express information for each annotation field, with obviously ramifications for accurate and efficient querying of *NeuroTransDB*. In an effort to standardize entries for different annotation fields specific controlled vocabularies were adopted during curation.

#### Age and gender normalization

We observed several different ways of representing age such as ‘24 yrs’, ‘25 yo’ and ‘23 ± 2 years old’. All age values were standardised by converting to simple decimal numbers, e.g. 24.00 for 24 years. Similarly for gender, we used a consistent representation of ‘M’ and ‘F’. As an example, gender information for GSE33528 samples were reported in the associated article (40) as ‘70% of the participants were women’. Here, we annotated the information as ‘70% female’. Although the annotations such as ranges (e.g. ‘23 ± 2 years old’), ratios (male/female: 19/9), or percentages (70% female) (40) are study-level annotations, they were provided as sample level annotations; as they do not contribute to reasonable cohort selection we did not normalize them.

#### Phenotype, brain region and stage normalization

Disease phenotype and stage information contributes to specific details of clinical manifestations whereas the tissue source (hereafter brain region) caters to the sample origin. For all the curated phenotype mentions (human), we generated a binning scheme: diseased, control or treated. These binned terms were further mapped to controlled vocabularies provided by Alzheimer’s Disease Ontology (ADO) (32). Other annotated terms that are not specific to AD were mapped to the Human Disease Ontology (33), Medical Subject Headings (MESH), Medicinal Dictionary for Regulatory Activities (MEDDRA) and Systematized Nomenclature of Medicine - Clinical Terms (SNOMED-CT) (34) ontologies (http://bioportal.bioontology.org/ontologies/MESH and http://bioportal.bioontology.org/ontologies/MEDDRA). This caters the need to query samples at a more abstract level, for downstream analysis. In total, for AD, we obtained 481 phenotype mentions assigned to at least one entry in the bins generated. Similarly, all the stage mentions (117 terms) were mapped to ADO, and ONTOAD (35). Mentions of brain region (41 unique terms) were tagged to Brain Region and Cell Type Terminology (BRCT) (http://bioportal.bioontology.org/ontologies/BRCT?p=summary). Please refer to Supplementary File S2 for detailed mapping of human annotation terms to controlled vocabularies.

#### Normalization of animal models

Similar to human phenotype normalization, we have normalized mouse and rat phenotype terms to EFO and SNOMED-CT. Different treatment procedures have been used to generate animal models that capture specific aspects of human diseases. At times, the incomplete nature of the models could lead to inadequate or misinterpretation of results. Thus, it is necessary to know the experimental procedures used on these animal models. To enhance this interpretation, we have binned all the captured animal model information, during the metadata curation, to a higher level of abstraction, further mapped to EFO, the National Cancer Institute Thesaurus (36), and the BioAssay Ontology (37). In addition, we mapped mouse and rat names to EFO, Jackson Laboratory database identifiers, and Sage Bionetworks Synapse Ontology (http://jaxmice.jax.org/query/f?p=205:1:0 and http://bioportal.bioontology.org/ontologies/SYN). This provides more flexibility during querying of samples from specifically treated animal models. Please refer to Supplementary Files S3 and S4 for mapping of mouse and rat-related terms to controlled vocabularies.

For some of the metadata terms, there were no controlled vocabularies  available, e.g.  ‘Vehicle #1:non- transgenic’ or ‘BDNF-treated’, describes that the mouse is non-transgenic and a vehicle in the former case, while in the second case it is specific gene treatment. Such terms were mapped to either of the phenotype’s controlled vocabulary. In case of human stage mentions, specific stages such as Braak II or cognitive scores, such as CERAD, MMSE, etc. could not be mapped to any staging controlled vocabulary as most of the ontologies used higher level of staging, namely Braak. Moreover, in most of the ontologies cognitive tests are not classified under staging, but rather as cognitive tests. This has prompted us to generate a more detailed hierarchical representation of the above-mentioned binning schemes, which will be published separately as ontology, specifically for neurodegenerative gene expression studies. However, for current version, we stick to the already available controlled vocabularies, in addition to our internal classification.

## Curation results and discussion

### Compliance to standards

**Figure 5.**
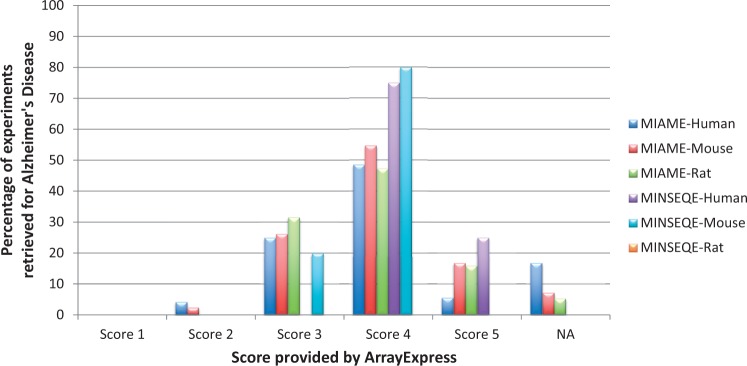
**Distribution of MIAME and MINSEQE scores for all automatically retrieved Alzheimer’s Disease gene expression experiments in ArrayExpress Database (for human, mouse and rat), as of December 2014.** Percentage is calculated as (total number of AD experiments with a certain score)/(total number of AD experiments). ‘NA’ are the experiments which were not present in ArrayExpress. These scores reflect adherence to compliance standards by the data submitters, needed for re-investigation and reproducibility. It is observed that large percentage of experiments fall under score 4, shows that the required minimum information is still incomplete. The list of experiment IDs along with their associated scores, used for generating this statistics are provided in Supplementary File S1.

Authors tend to provide minimum information as required by the guidelines in archives; publishing major part of the experimental metadata annotations in associated publication. To test, whether the authors adhere to the minimum compliant standards, we performed an assessment of the complaint scores provided by ArrayExpress, the highest score being 5, for Alzheimer’s studies. Figure 5 shows the trend in distribution of retrieved AD experiments (see Data Retrieval section) in ArrayExpress, based on the published MIAME and MINSEQE scores (for human, mouse and rat experiments). We observe the trend of submission is concentrated around the score of 4, showing that the submitted data are not fully MIAME or MINSEQE compliant; leading to variable levels of information stored in these archives.

To conclude that not all the submitters' abide 100% by the compliant standards, we investigated if this trend is same for all other disease domains; we chose one among the most studied cancer disease, Lung Cancer, and generated similar results to AD. Supplementary Figure S5 shows the distribution of compliant standards across Lung Cancer studies. From this observation, we show that the loss of information follow the same pattern across all submissions (varying mostly around score of 4). As a result, automated retrieval and meta-analysis is impeded, due to lack of information availability. Details of the experiment IDs investigated for AD and Lung Cancer, along with compliant scores is provided in Supplementary File S1.

### Retrieval and prioritization of indication specific studies from GEO and ArrayExpress

Retrieval of experiment IDs using a keyword search (*cf. *Data Retrieval section) also acquires false positive experiments. Any non-disease specific experiment performed by an author named ‘Alzheimer’ is also retrieved when searching for AD specific experiments. For example, E-MTAB-2584 aims to investigate neuronal gp130 regulation in mechanonociception but was retrieved for AD since one of its author’s name is Alzheimer. Moreover, we also obtained experiments for related diseases such as Epilepsy, or Breast Cancer, when querying for AD. For example, GSE6771, and GSE6773 are Epilepsy studies; GSE33500 belongs to Nasu Hakola Disease; all these studies were retrieved when queried for Alzheimer. Incorrect organism specificity was also noticed during prioritization. For example, GSE5281 was retrieved as rat study although it belonged to human. Similarly, GSE2866 was retrieved as mouse study but it belonged to zebra fish. Although incorrectly identified studies are not too high, this still indicates the need to include organism and disease specificity filter during prioritization. Additionally, we manually identified a few experiments that were not retrieved using these keywords, which were also included in the database.

**Figure 6.**
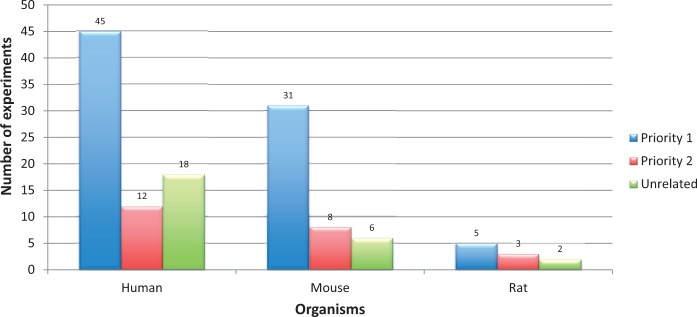
**Priority classification statistics for Alzheimer’s disease gene expression experiments retrieved from ArrayExpress and GEO (for human, mouse and rat).** Alzheimer’s disease experiments were retrieved using keywords. Applying the Experiment Prioritization guidelines, they were manually classified to one of the priority classes. Among them, 20% of the experiments were not related to Alzheimer’s disease. The digits on the bars represent number of experiments.

Further on, just by applying these two filter criteria does not assure that all retained experiments were specific to AD. For example, there could be some experiments that aim at a certain pathway that are also relevant in the area of neurodegeneration, but the experiment submitted to the repository does not deal with AD pathology. As a consequence, additional disease relevancy conditions were included before prioritization (*cf.* Experiment Prioritization section). An overview of all the retrieved AD experiments, categorized to one of the priority classes is shown in Figure 6. In addition, a list of priority 1 experiments (for human, mouse and rat) is provided in Supplementary file S5. This figure indicates that nearly 20% of the retrieved studies are in any case not related to AD. On the other hand, to identify the remaining 80% of the experiments (prioritized as 1 and 2) we need massive manual filtering by trained personnel. Only if the archives take an initiative to apply such a structured classification for all uploaded experiments, individual time-cost can be reduced to a greater extent.

Some experiments contain cell lines or other disease samples in addition to Alzheimer’s patient samples. Experiment GSE26927 additionally contain samples from patients suffering from Parkinson’s disease, multiple sclerosis, etc. To be able to query only AD related samples for integrative analysis, we additionally included priority information at sample level. For example, we tagged Alzheimer’s disease samples to AD1 whereas multiple sclerosis samples to MS1. Please refer to the README.txt file for various priority notations used.

### Metadata curation

**Figure 7.**
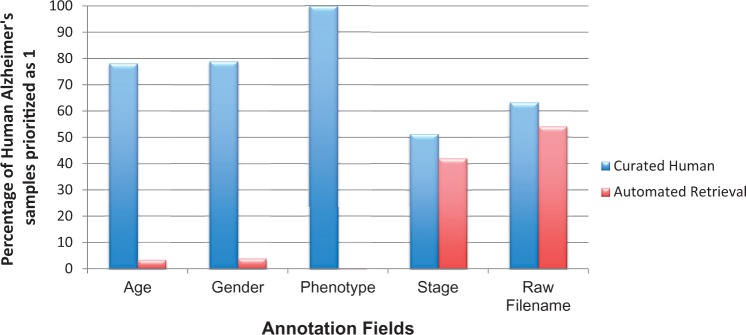
**Coverage of basic metadata annotation fields for human AD priority 1 samples with automated retrieval and manual curation.** Automated retrieval involved downloading the metadata information from ArrayExpress and GEO, programmatically. For missing meta-annotations, we applied manual curation step to harvest information from the published articles and their associated Supplementary materials. It is clear from the above statistics that manual curation accuracy for basic annotations, such as patient’s clinical manifestations, and raw file information, is highly dependent on data availability.

The underlying metadata information for any gene expression study has been underrepresented and thus is largely under-utilized. To perform large-scale analysis, associated annotations are of utmost importance. With the availability of detailed annotation information, one is capable of selecting studies that focus on a particular attribute, such as stage or gender. Each priority class has a specific set of fields for curation; some fields are organism dependent. After prioritization of experiments (*cf.* Experiment Prioritization section), we expect to have ∼100% coverage of essential clinical and relational parameters during manual metadata curation for priority 1 studies. For example, age, gender, phenotype and stage are basic experimental variables for human studies. Additionally, in case of animal models, mouse and rat strain names are important for translational pipelines, as some strains are highly specific models for human NDD while others not (38). Irrespective of the organisms, samples mapped to their corresponding raw file identifiers are vital for running large-scale analysis. However, as shown in Figure 7, this does not hold true for human studies. From Figure 7, it is evident that even after performing thorough curation, we cannot achieve 100% in capturing information for these five basic metadata fields, a fact that is largely due to patient data privacy regulations. Similar is the case with mouse and rat information, see Supplementary Figure S6. Moreover, information related to animal models are much more scare, obstructing automated retrieval. Hence, manual curation accuracy is highly dependent on information availability, as curators cannot harvest information for annotation fields that are not available. On the contrary, the level of detail also depends on the type or aim of the experiment carried out. The authors and database owners obviously need to focus on the qualitative aspect of the experimental information, especially the phenotype of the sample, to allow normalized access for beginners, with standard prose, in order to support a robust computational analysis across all studies in ArrayExpress and GEO.

**Figure 8.**
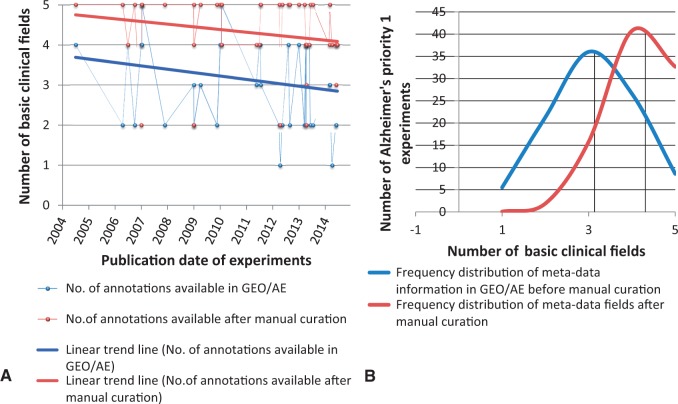
**Frequency distribution and Trend Analysis of human priority 1 Alzheimer’s disease gene-expression experiments for availability of five basic annotation fields in GEO/ArrayExpress sample page versus manual curation.** The five basic annotations considered here are age, gender, stage, phenotype and raw filename. (**A**) Red and blue line represents the linear trend analysis of the availability of meta-annotations for experiments (represented as dots) over years, which has declined. (**B**) The black line represents mean value of the number of annotation fields filled. It is evident from the shift in mean of the distribution analysis that manual curation plays a very important role in capturing the missing metadata information.

We selected five of the most common metadata fields (common to any disease domain such as age, gender, phenotype, stage and raw filename) and carried out a trend analysis of information availability versus time. Figure 8 (A) shows the trend over time for the metadata information provided in the archives versus the number of annotation fields that can be harvested after manual curation for human AD priority 1 experiments. Although a bit obscure, we can observe that the level of information submitted to the databases remains almost stable in the last decade (between 2 and 4 metadata fields). Moreover, with manual curation support, we were able to capture the majority of the remaining metadata from associated publications, Figure 8 (B) shows the shift in the mean value of the metadata availability. However, the trend is recently declining since the authors submit relatively lesser level of detailed information than in former times in the associated publications.

The incompleteness of metadata annotations contributed to a substantial increase in curation workload through an increased need for publication reading. This leads to a steep increase of the cost of the trained personal for curation. Overall, for the prioritization and metadata curation of AD gene expression studies, we spent about 1 year of four biocurators effort (working 10 h/week). This does not include the expert’s effort, who constantly provided guidance and monitored the curation work during the same duration.

### Accessing *NeuroTransDB*

Metadata annotations for priority 1 AD gene expression studies for human, mouse and rat organisms, from GEO and ArrayExpress, are stored as MySQL tables separately; downloadable as dump files at Fraunhofer SCAI File Transfer Protocol (FTP) website: http://www.scai.fraunhofer.de/NeuroTransDB.html. Please refer to the README.txt for details of how to install and use MySQL dumps. Additionally, these tables are provided as Excel files to allow users to use the curated information in their preferred tools/interface. Currently, the data is in its non-normalized form. Normalized data, tagged with standard ontologies (*cf.* Normalization of Metadata Annotations section), will be made available through the AETIONOMY Knowledge Base. Currently, we have provided human priority 1 studies normalized using our internal binning scheme. Half yearly updates are planned. Our ultimate goal is to make *NeuroTransDB* a comprehensive resource for researchers working on large-scale meta-analysis in the field of neurodegenerative diseases.

## Conclusion and future directions

*NeuroTransDB *fills the gap for large-scale meta-analysis on publicly available gene-expression studies in the field of neurodegeneration. It joins bits of missing metadata information, scattered in public archives and associated publications, into a consistent, easily accessible and regularly updated data resource. Additionally, in this paper, we have systematically specified key issues encountered during selection of relevant gene expression studies from public archives, along with their associated metadata information. We observed a huge lack of structured metadata in these archives, hampering automated large-scale reusability on a usable level of abstraction. We present here recommendations, as guidelines, for prioritizing relevant studies and a step-by-step protocol for metadata curation. The challenges faced in the course of the development of these guidelines have been pointed out, and the huge manual effort has been made explicit.

The work presented here has listed metadata fields, which have been generated based on disease expert consultation. They are highly important for choosing the right subsets of expression studies to answer complex biological questions underlying a diseased pathology. Some additional fields are included for animal models studies to allow maximal use for translational research. For all the manually curated fields, we describe normalization strategies in an attempt to provide standards for more robust automated querying and interoperability. Our results show the amount of information that is scattered in various resources, requiring extensive manual effort to capture the same. Additionally, we report that even with comprehensive manual harvesting, we were not able to capture 100% of information to fill for the basic annotation fields. We demonstrate convincingly that data availability depends largely on the meticulousness of the submitters. Additionally, it also depends on the aim of the experiment carried out. On an average, considering all the retrieved AD experiments, the submitters provide about 60% of the most basic metadata information. The outlined guidelines could be of significant value to other researchers working on gene-expression studies. The described key issues we faced during such a curation work could influence the data submission and data storage architecture of public repositories.

Subsequently, we plan to extend the curation pipeline to other NDD diseases namely, Huntington’s disease. A more gene-expression specific ontology will be built based on the curated annotations for selecting a subset of studies for meta-analyses. Although, microarray studies are the major contributors to the public repositories, RNA-Seq data are rapidly growing. We comprehend that it will be necessary for us to identify all the relevant RNA-Seq studies, since their large storage space has contributed to disperse nature of the available raw data.

## Supplementary Data

Supplementary data are available at *Database* Online.

Supplementary Data
