# Supplementary material for: NeuroTransDB: highly curated and structured transcriptomic metadata for neurodegenerative diseases
Source: Database (Oxford). 2015 Oct 15;2015:bav099. doi: 10.1093/database/bav099 (PMC4608514; doi:10.1093/database/bav099)
Supplement: Supplementary Data [file supp_2015_bav099_index.html]

Supplementary Data 

# *NeuroTransDB*: highly curated and structured transcriptomic metadata for neurodegenerative diseases

## Supplementary Data

files

- Supplementary Data - zip file
