# Supplementary material for: NeuroTransDB: highly curated and structured transcriptomic metadata for neurodegenerative diseases
Source: Database (Oxford). 2015 Oct 15;2015:bav099. doi: 10.1093/database/bav099 (PMC4608514; doi:10.1093/database/bav099)
Supplement: Supplementary Data [file supp_bav099_supplementary_data.zip › CurationGuidelines.pdf]

FRAUNHOFER-INSTITUTE FOR ALGORITHMS AND SCIENTIFIC COMPUTING (SCAI)

Meta-Data Curation Guidelines of Publicly Available Gene-expression Data

Fraunhofer
SCAI

Short description

This document describes a guideline for annotating Microarray metadata for datasets coming from public repositories like Gene Expression omnibus (GEO) and ArrayExpress(AE).

The relevant fields were described with the help of disease experts. This document describes the guidelines of how to annotate each field and where to find the relevant information. Some possible examples harbouring each field is also provided.

Currently, only Alzheimer's Disease experiments are considered. Each experiment is curated by two annotators to obtain good inter-annotator consistency.

What each term means?

**Term**	**Meaning**
Field	Field is the column name in *NeuroTransDB*. Each field represents different kind of data obtained through curation
Description	Describes what data has to be filled in each of the fields concerned
Example	Examples of data filled in each field
GUIDELINE	Describes the importance of the field
SOURCE	Where to obtain the information

Databases concerned

Database Name	URL	Maintained by
ArrayExpress (AE)	https://www.ebi.ac.uk/arrayexpress/	Europe
GEO (Gene Expression Omnibus)	http://www.ncbi.nlm.nih.gov/geo/	United States of America

*Characters given in **BOLD** are important

Field	series_iid
Description	Experiment ID from GEO or ArrayExpress
Examples	ArrayExpress: **E-GEOD-**9990, **E-MTAB-**28, **E-TABM-**726, **E-MEXP-**1028
	GEO: **GSE**9990
GUIDELINE	An ArrayExpress experiment ID or GEO experiment ID serves as unique identifier for given experiment and is most easy way to unambiguously access any experiment from ArrayExpress or GEO. This information has been automatically downloaded.
SOURCE	Image on Left (AE): https://www.ebi.ac.uk/arrayexpress/experiments/E-MTAB-28/<br>Image on Right (GEO): http://www.ncbi.nlm.nih.gov/geo/query/acc.cgi?acc=GSE1

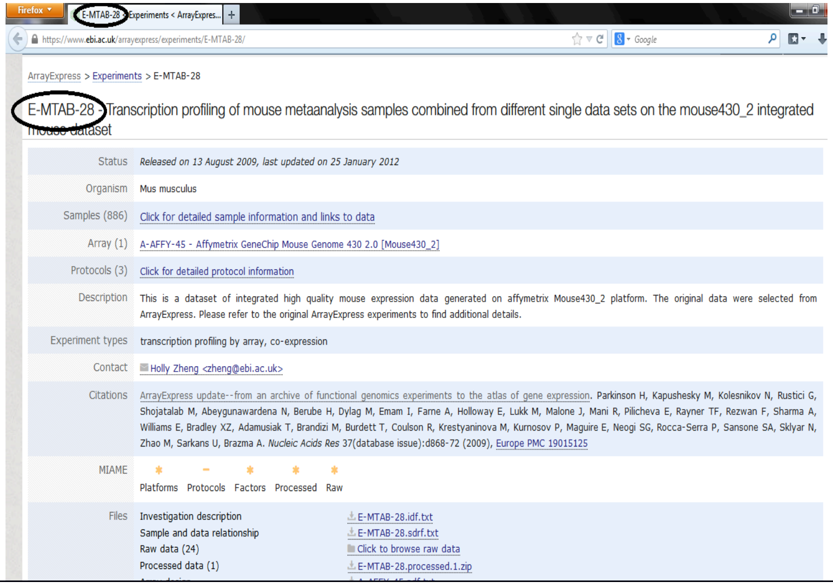

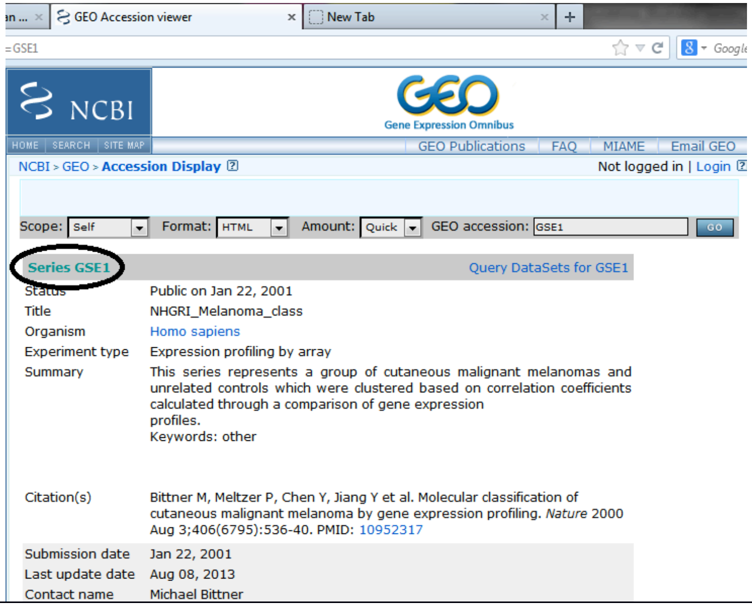

Field	sample_id
Description	Sample ID of the samples used in Experiment/Series in ArrayExpress/GEO respectively
Examples	ArrayExpress: **GSM**32049
	GEO: **GSM**32054
GUIDELINE	ArrayExpress sample ID's or GEO sample ID's serve as unique identifier for the given samples from an Experiment and these ID's are the most easy way to unambiguously access any sample from ArrayExpress or GEO databases. This information has been automatically downloaded.
SOURCE	Image on Left (AE): https://www.ebi.ac.uk/arrayexpress/experiments/E-GEOD-1829/samples/<br>Image on Right (GEO): http://www.ncbi.nlm.nih.gov/geo/query/acc.cgi?acc=GSE1829

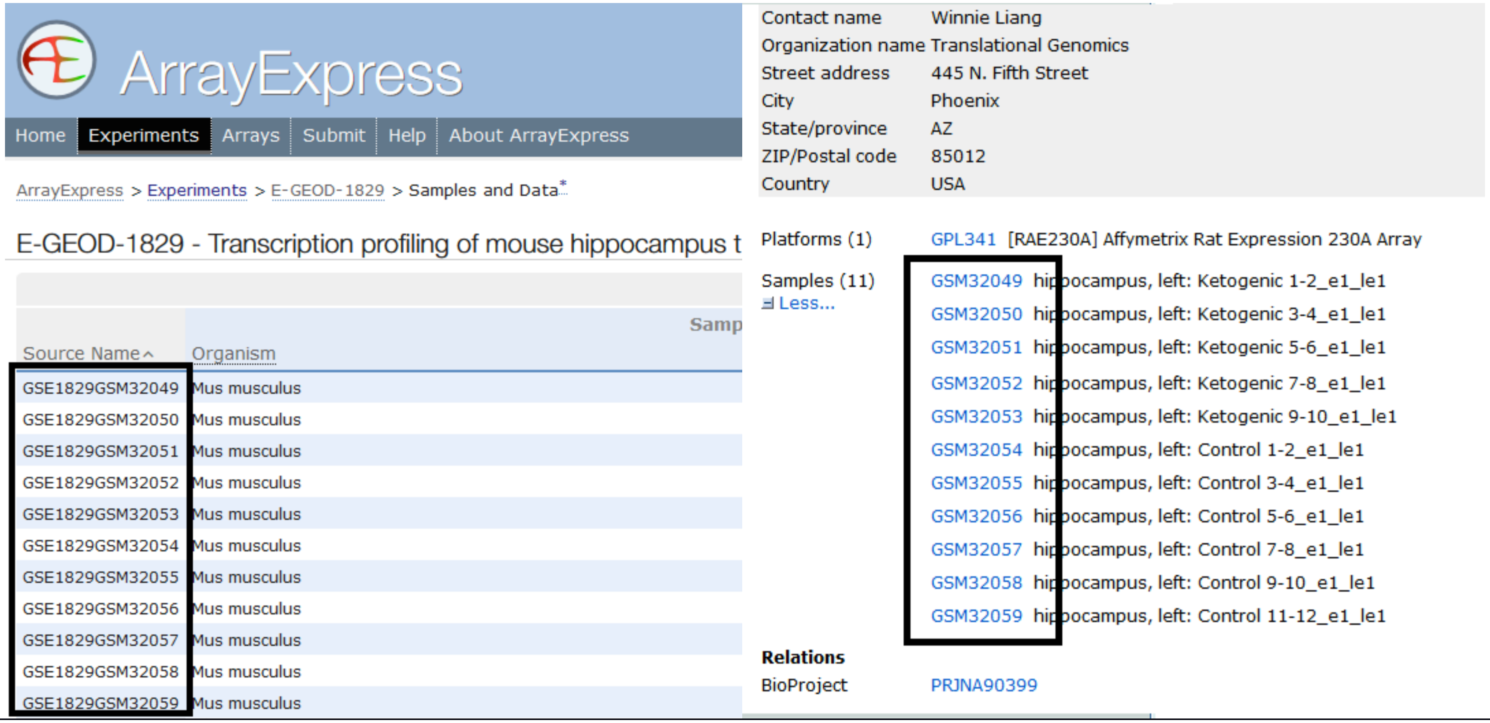

Fraunhofer
SCAI

<table>
<tr><td>Field</td><td>sample_title</td></tr>
<tr><td>Description</td><td>Title of the individual sample in GEO or ArrayExpress. Generally title information from ArrayExpress cannot be found directly.</td></tr>
<tr><td rowspan="2">Examples</td><td>ArrayExpress:</td></tr>
<tr><td>GEO: Severe 701</td></tr>
<tr><td>GUIDELINE</td><td>Title may contain information about disease state of the sample, if the sample is a replicate, or a mutant. This information has been automatically downloaded, however it is rechecked manually for correctness.</td></tr>
<tr><td>SOURCE (WHERE TO FIND INFORMATIO N)</td><td>Image on Below (AE): https://www.ebi.ac.uk/arrayexpress/experiments/E-GEOD-36232/samples/
Image on Above (GEO): http://www.ncbi.nlm.nih.gov/geo/query/acc.cgi?acc=GSE1375
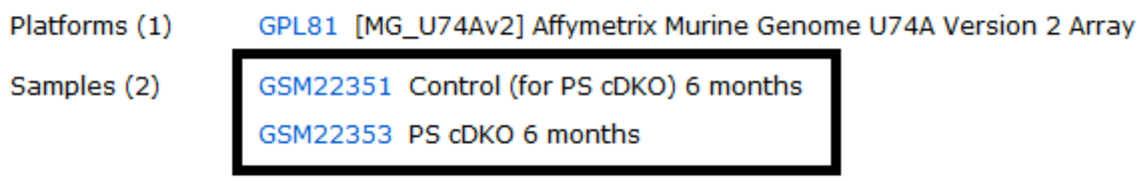

Title information from AE can be found by clicking FastQ (click the link given for AE above)
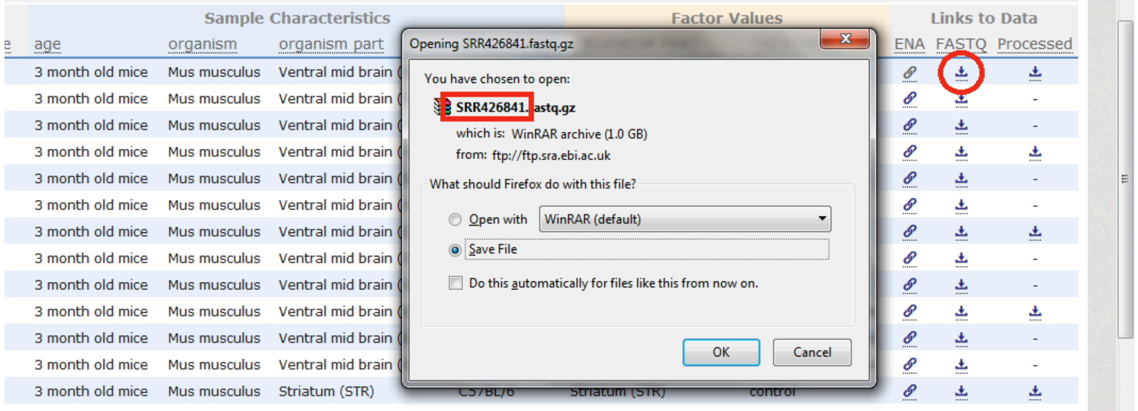

</td></tr>
</table>

Field	sample_type
Description	Biomolecules extracted from samples for analysis in Experiments
Examples	ArrayExpress: low-molecuar-weight RNA derived from total RNA
	GEO: RNA, total RNA, SRA, genomic DNA, pooled RNA from nine control mice/rat, Single RNA
GUIDELINE	The entity extracted from organism/cells are proteins or nucleic acids or in special case SRA
SOURCE	Image on Left (GEO): http://www.ncbi.nlm.nih.gov/geo/query/acc.cgi?acc=GSM884353<br>Image on Right (AE): http://www.ebi.ac.uk/arrayexpress/experiments/E-GEOD-48028/samples/<br>Mostly you can find information about the “type” from GEO sample files but for exact information one must read paper and/or examine ArrayExpress sample data file sheet. There are experiments where in one single sample, RNA’s are pooled from many pateints/organisms, in that cases “type” should be “pool from ‘n-number’ mice/rat/patients” or just pool. In case of SRA (Sequence Read Archive) one can enter “SRA:total RNA” in “type” field (as shown in figure). Consider experiment E-GEOD-48028 where sample “type” was found to be “RNA” in GEO, but AE had more definite and precise information about the “type” i.e. “low-molecuar-weight RNA derived from total RNA” was found. If this information has been automatically downloaded, then it is rechecked manually for correctness.

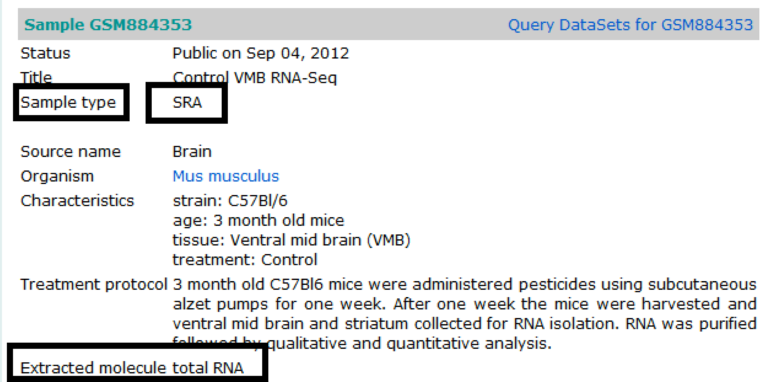

Sample GSM884353	Query DataSets for GSM884353
Status	Public on Sep 04, 2012
Title	Control VMB RNA-Seq
Sample type	SRA
Source name	Brain
Organism	Mus musculus
Characteristics	strain: C57Bl/6<br>age: 3 month old mice<br>tissue: Ventral mid brain (VMB)<br>treatment: Control
Treatment protocol	3 month old C57Bl6 mice were administered pesticides using subcutaneous alzet pumps for one week. After one week the mice were harvested and ventral mid brain and striatum collected for RNA isolation. RNA was purified followed by qualitative and quantitative analysis.
Extracted molecule	total RNA

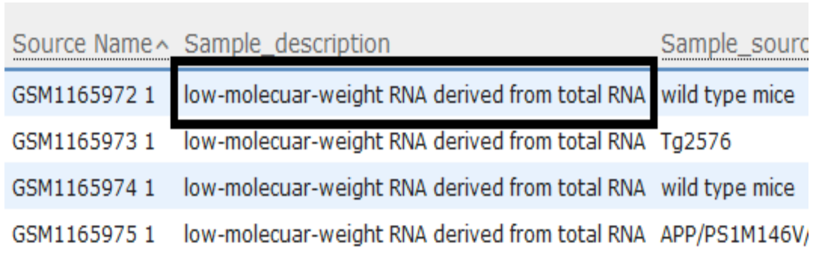

Source Name	Sample_description	Sample_sourc
GSM1165972 1	low-molecuar-weight RNA derived from total RNA	wild type mice
GSM1165973 1	low-molecuar-weight RNA derived from total RNA	Tg2576
GSM1165974 1	low-molecuar-weight RNA derived from total RNA	wild type mice
GSM1165975 1	low-molecuar-weight RNA derived from total RNA	APP/PS1M146V/

Field	disease_keyword
Description	Keyword used during data retrieval
Examples	ArrayExpress: Alzheimer
	GEO: Alzheimer
GUIDELINE	The keyword used for retrieval
SOURCE	This information is mostly automatically mined out

Field	domain_specificity
Description	If the experiment or rather sample is relevant to the disease keyword used for retrieval
Examples	This field takes binomial values. “Yes” if experiment is related to disease domain else “No”
GUIDELINE	Gives information about the relevance of sample to the disease being looked up. Relevance means that the experiment is substantially related to Alzheimer’s diseases. It’s a Boolean value either YES or NO. This has helped us improve our data retrieval method by improving our keyword (synonym) list
SOURCE	Paper, GEO or ArrayExpress description page

Field	experiment_title
Description	Title of the experiment, which is downloaded automatically
Examples	For experiment GSE12685, the title provided by two databases are:<br>ArrayExpress: Transcription profiling of human Alzheimers disease patients to identify genes regulating synaptic function and neuroplasticity in incipient AD
	GEO: Expression of mRNAs Regulating Synaptic Function and Neuroplasticity in Incipient AD
GUIDELINE	The title is automatically retrieved from the databases
SOURCE	This information is mostly automatically mined out

Fraunhofer
SCAI

Field	age
Description	Age of the source from which the sample is retrieved. Additionally, age of the cells is also included if cells are used in an experiment. If available information about age at death or age at disease onset should also be included
Examples	Age organism: 6 months, 6 mon, Post Natal Day 15, P19, 24 - 28 weeks, 6-8 wks, 3d, age 540 days, Gestation day 15-16, 16.5-day-old, 12 months, age at onset: 25yr/age at death: 74yrs
	Age cell: 15 days, age of culture day 5
GUIDELINE	Microarray analysis reveals age-related differences in gene expression and hence inclusion of age data is vital
	Image on Left (GEO): http://www.ncbi.nlm.nih.gov/geo/query/acc.cgi?acc=GSM26703<br>Image on Right (AE): https://www.ebi.ac.uk/arrayexpress/experiments/E-GEOD-36232/samples/<br>Age information can either be explicitly mentioned in AE/GEO or have to be searched from research paper linked to experiment. Age information is usually found in Materials and Methods section of paper, figure or supplementary files provided in research paper. One can find cell age information from image on Left. Sometimes age information is given in the form of range. If age information was automatically retrieved we check again if it is correct.

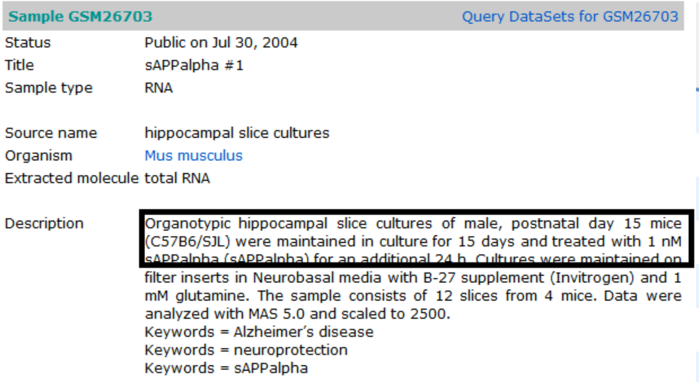

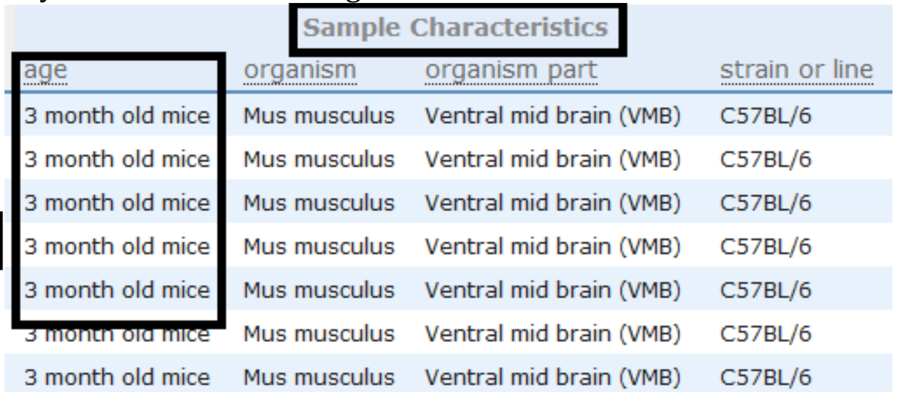

Field	gender
Description	Gender of the sample organism
Examples	Male, Female, f, m, Homo, Mixed, Other, (pooled age information is given as x% male and y% female (x = numerical value, y = numeric value and x+y = 100)
GUIDELINE	Gender data is used to analyse gender biased or gender specific gene expression analysis and hence gender information is vital
SOURCE	Image reference: http://www.ncbi.nlm.nih.gov/pmc/articles/PMC3341364/<br>Gender information can either be explicitly mentioned in AE/GEO or have to be searched from research paper linked to experiment. Gender information is usually found in Materials and Methods section of paper, figure or supplementary files provided in research paper. If gender information was automatically generated we check if it is correct.<br>Consider the ID E-GEOD-36232 where gender information was not available in GEO and AE but was found from paper.

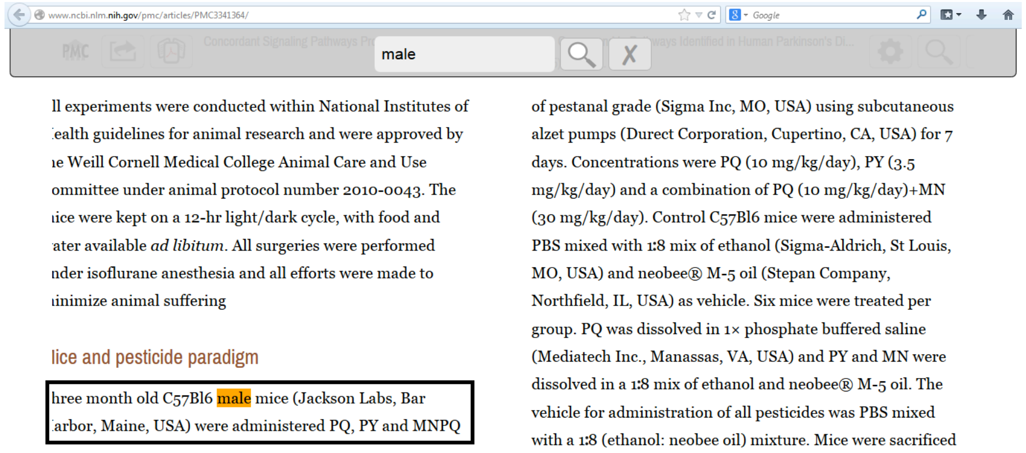

Field	age_info_section
Description	Page or section where the information about the age was found
Examples	http:// or sdrf or ftp://
GUIDELINE	Serves as a reference link to literature information from Paper or AE/GEO
SOURCE	Address bar of the web page where you found age information or in case of downloaded supplementary files one can right click the hyperlink of "supplementary file" -> Copy link location and paste in the "age_info_section" field. If age information was found from AE or GEO web pages, insert the specific URL from the AE/GEO sections

Field	gender_info_section
Description	Page or section from where gender information about the sample was found
Examples	http:// or sdrf or ftp://
GUIDELINE	Serves as a reference link to literature information from Paper or AE/GEO
SOURCE	**Address bar** of the web page where we found gender information or in case of downloaded supplementary files one can right click the hyperlink of "supplementary file" -> Copy link location and paste in the "gender_info_section" field. If Gender information was found from AE or GEO web pages, insert the specific URL from the AE/GEO sections.

Field	series_id
Description	The experiment IDs as downloaded from the databases
Examples	GSE12685 or E-GEOD-12685
GUIDELINE	Identifier of the experiment
SOURCE	Series webpage of GEO

Field	source_database
Description	Name of the database from which the experiment's information was downloaded
Examples	GEO or ArrayExpress
GUIDELINE	The data origin information

Field	geo_series_id
Description	The experiment IDs from GEO
Examples	GSE12685
GUIDELINE	Since most of the experiments are firstly downloaded from ArrayExpress, the GEO experiment IDs are represented as E-GEOD-12865. However, we automatically revert the series ID here and save the original ID from GEO
SOURCE	Series webpage of GEO

Field	original_sample_id
Description	The sample Ids downloaded as is from the databases
Examples	For experiment E-GEOD-12685, an example sample id from ArrayExpress is GSE12685GSM318211
GUIDELINE	ArrayExpress modifies the sample IDs from GEO. For example, in GSE12865 the sample ID is GSM318211. However, ArrayExpress refers it as GSE12685GSM318211.
SOURCE	Sample webpage of GEO

Field	phenotype
Description	Phenotype of the sample
Examples	Diseased_AD, Diseased_PD, Diseased_EP, normal, mutant, control, normal_elderly_control, young_control, AD_control, KO mice/rat, KI mice/rat, Treated vs non treated mice/rat, 3xTg, 5x backcrossed mice/rat, pool young, pool adult, Sham mice/rat
GUIDELINE	This field describes the observable characteristics or traits that can distinguish between normal and diseased patient/cell
SOURCE	Phenotype information is either available in title of the sample or have to be searched in AE or GEO files. If you do not find such information in ArrayExpress or GEO files a final check should be done in research paper and specifically in diagrams, graphs, tables and supplementary materials given in research paper.<br><br>Image on Left (GEO): http://www.ncbi.nlm.nih.gov/geo/query/acc.cgi?acc=GSE6771<br>Image on Right (AE): https://www.ebi.ac.uk/arrayexpress/experiments/E-GEOD-5281/samples/

Platforms (1) GPL4757 Ion Channel Splice Array

Samples (10) Less...

GSM156001	Temporal Cortex Control (mTLE) 1
GSM156003	Temporal Cortex Control (mTLE) 3
GSM156051	Temporal Cortex Control (mTLE) 2
GSM156052	Temporal Cortex Control (mTLE) 4
GSM156159	Temporal Cortex Control (mTLE) 5
GSM156160	Temporal Cortex Control (mTLE) 6
GSM156161	Temporal Cortex Control (mTLE) 7
GSM156162	Temporal Cortex Control (mTLE) 8
GSM156164	Temporal Cortex Control (mTLE) 9
GSM156169	Temporal Cortex Control (mTLE) 10

Sample_source_name	Organism	age	bio-source name	cell type
brain, Entorhinal Cortex	Homo sapiens	63 years	EC control 1	layer III neurons
brain, Entorhinal Cortex	Homo sapiens	85 years	EC control 2	layer III neurons
brain, Entorhinal Cortex	Homo sapiens	80 years	EC control 3	layer III neurons
brain, Entorhinal Cortex	Homo sapiens	80 years	EC control 4	layer III neurons
brain, Entorhinal Cortex	Homo sapiens	102 years	EC control 5	layer III neurons
brain, Entorhinal Cortex	Homo sapiens	79 years	EC control 6	layer III neurons
brain, Entorhinal Cortex	Homo sapiens	76 years	EC control 7	layer III neurons
brain, Entorhinal Cortex	Homo sapiens	83 years	EC control 8	layer III neurons
brain, Entorhinal Cortex	Homo sapiens	79 years	EC control 9	layer III neurons
brain, Entorhinal Cortex	Homo sapiens	88 years	EC control 10	layer III neurons
brain, Entorhinal Cortex	Homo sapiens	82 years	EC control 11	layer III neurons
brain, Entorhinal Cortex	Homo sapiens	69 years	EC control 12	layer III neurons
brain, Entorhinal Cortex	Homo sapiens	78 years	EC control 13	layer III neurons
brain, hippocampus	Homo sapiens	85 days	HIP control 1	layer III neurons
brain, hippocampus	Homo sapiens	80 years	HIP control 2	layer III neurons
brain, hippocampus	Homo sapiens	80 years	HIP control 3	layer III neurons
brain, hippocampus	Homo sapiens	102 years	HIP control 4	layer III neurons
brain, hippocampus	Homo sapiens	63 years	HIP control 5	layer III neurons
brain, hippocampus	Homo sapiens	79 years	HIP control 6	layer III neurons
brain, hippocampus	Homo sapiens	76 years	[illegible]	layer III neurons

Field	phenotype_info_section
Description	Page or section from where phenotype information about the sample was found
Examples	http://www.ncbi.nlm.nih.gov/geo/query/acc.cgi?acc=GSE32534
GUIDELINE	Serves as a reference link to literature information from Paper or AE/GEO
SOURCE	**Address bar** of the web page where you found phenotype information **or** in case of downloaded supplementary files one can right click the hyperlink of "supplementary file" -> Copy link location and paste in the "phenotype_info_section" field. If phenotype information was found from AE or GEO web pages, insert the specific URL from the AE/GEO sections.

Field	stage
Description	This field describes disease stage of AD
Examples	onset(SNCA inclusion bodies), Braak1 stage according to Braak and Braak/mmse score-15
GUIDELINE	Provides information on the disease progression. It can be name of the stage (mild/severe) or values of different scoring system (mini-mental state examination score/Hoehn and Yahr's Staging score). If both are available, we include them as is. It is crucial to mention the criterion behind the staging.

Field	stage_info_section
Description	Page or section from where phenotype information about the sample was found
Examples	http://
GUIDELINE	Serves as a reference link to literature information from Paper or AE/GEO
SOURCE	**Address bar** of the web page where you found stage information **or** in case of downloaded supplementary files one can right click the hyperlink of "supplementary file" -> Copy link location and paste in the "stage_info_section" field. If phenotype information was found from AE or GEO web pages, insert the specific URL from the AE/GEO sections.

Field	replicate_type
Description	If the sample is a replicate, then what type it is: biological or technical
Examples	Technical
GUIDELINE	This supports the meta-analysis
SOURCE	Replicate information can either be explicitly mentioned in AE/GEO or have to be searched from research paper linked to experiment. This information is usually found in Materials and Methods section of paper, figure or supplementary files provided in research paper.

Field	replicate_of
Description	If the sample is a replicate, then provide the id of its replicate
Examples	GSM969416
GUIDELINE	This supports the meta-analysis
SOURCE	Replicate information can either be explicitly mentioned in AE/GEO or have to be searched from research paper linked to experiment. This information is usually found in Materials and Methods section of paper, figure or supplementary files provided in research paper.

Field	sample_id
Description	The sample Ids resolved to the correct names in GEO
Examples	For experiment E-GEOD-12685, an example sample id from ArrayExpress is GSE12685GSM318211. This is resolved to GSM318211
GUIDELINE	ArrayExpress modifies the sample IDs from GEO. For example, in GSE12865 the sample ID is GSM318211. However, ArrayExpress refers it as GSE12685GSM318211.
SOURCE	Sample webpage of GEO

Field	pmd
Description	PMD= Postmortem duration. Duration between the death of the patient (if dead) and extraction of the sample.
Examples	2 min or 14 hrs
GUIDELINE	Postmortem duration/ Postmortem interval determine the RNA integrity of the extracted sample.
SOURCE	Image reference left (Research Paper): http://www.ncbi.nlm.nih.gov/pmc/articles/PMC3784285/<br>Image on right (AE): https://www.ebi.ac.uk/arrayexpress/experiments/E-GEOD-6774/samples/<br><br>PMD/ PMI information can either be explicitly mentioned in AE/GEO or have to be searched from research paper linked to experiment. This information is usually found in Materials and Methods section of paper, figure or supplementary files provided in research paper. Consider ID E-GEOD-47038 for which pmd was neither found in GEO/AE after 2 checks but was later found after reading corresponding paper for the experiment.

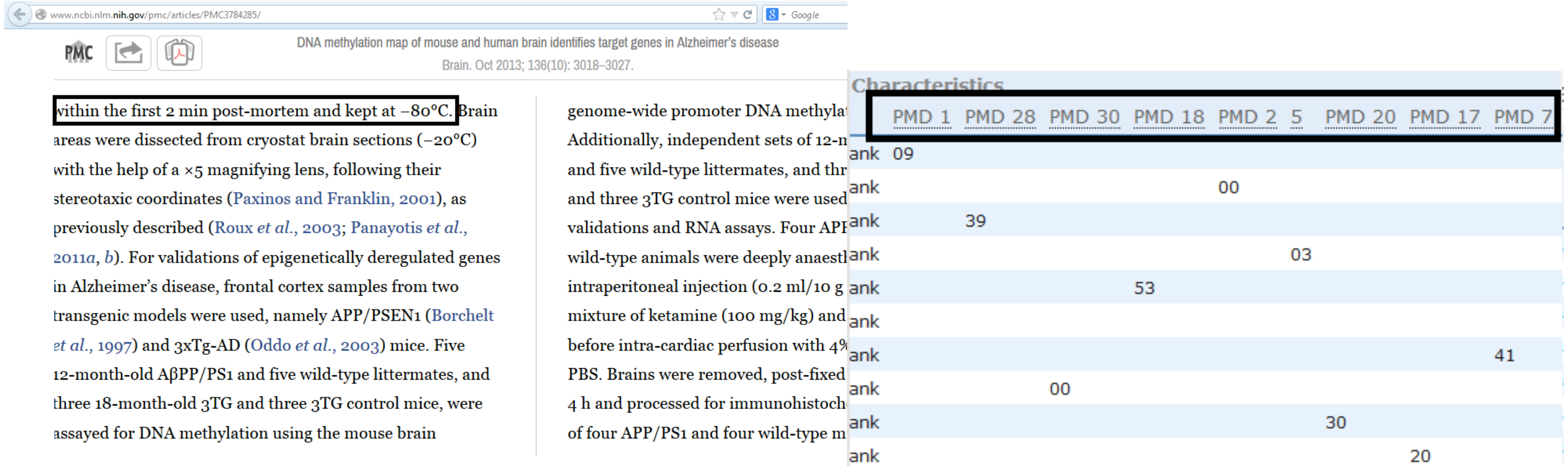

Fraunhofer SCAI

Field	ph_value
Description	pH value of the sample at the time of analysis.
Examples	4
GUIDELINE	It determines the sample source quality
SOURCE	pH information can either be explicitly mentioned in AE/GEO or have to be searched from research paper linked to experiment. This information is usually found in Materials and Methods section of paper, figure or supplementary files provided in research paper.

Field	cause_of_death
Description	Describes the reason for death of the source from which the sample was extracted
Examples	Heart attack
GUIDELINE	This helps us to determine if AD is the cause of death or other/comorbid diseases
SOURCE	This information is mostly mentioned in the associated research papers linked to experiment. This information is usually found in Materials and Methods section of paper, figure or supplementary files provided in research paper.

Field	comorbidity
Description	Describes the co-existence of other diseased
Examples	Diabetes Mellitus
GUIDELINE	This helps us to determine if AD is a side effect or vice versa of other co-existing diseases
SOURCE	This information is mostly mentioned in the associated research papers linked to experiment. This information is usually found in Materials and Methods section of paper, figure or supplementary files provided in research paper.

Field	priority
Description	Describes the priority of the experiment after the classification step
Examples	AD1
GUIDELINE	This is the Priority classification information
SOURCE	Manually carried out by expert curators. Please refer to the paper for guidelines

Field	sample_priority
Description	Describes the priority of the sample identified during meta-curation
Examples	AD1
GUIDELINE	Some experiments contain samples that are derived from humans/animals and/or cell lines. In other cases the samples are from different diseases such as Breast Cancer. To improve the retrieval method we have additionally assigned priority to individual samples
SOURCE	Manually carried out by expert curators. Please refer to the paper for guidelines

Field	raw_file_location
Description	URL to the raw file provided by databases
Examples	http://www.ncbi.nlm.nih.gov/geo/download/?acc=GSE12685&format=file
GUIDELINE	This provides us information as to where to download raw files
SOURCE	GEO or ArrayExpress series webpage

Field	cell_types
Description	Types of cells used as samples
Examples	primary hippocampal neurons, primary mouse embryonic fibroblasts (MEFs), DA neurons, non-DA neurons and glia cells
GUIDELINE	Important for gene expression analysis and difference in gene expression in different cell types
SOURCE	Image on Left (GEO http://www.ncbi.nlm.nih.gov/geo/query/acc.cgi?acc=GSM512701<br>Image on Right (AE): https://www.ebi.ac.uk/arrayexpress/experiments/E-GEOD-36232/samples/<br><br>Cell type information can either be explicitly mentioned in AE/GEO or have to be searched from research paper linked to experiment. This information is usually found in Materials and Methods section of paper, figure or supplementary files provided in research paper.<br><br>Channel 1<br>Source name: Mouse primary hippocampal neurons, PBS control<br>Organism: Mus musculus<br>Characteristics: cell type: primary hippocampal neurons<br>strain: C57BL/6<br>developmental stage: E16.5<br>treatment: PBS control<br><br>**Results** Top<br>**HtrA2 KO cells show increased sensitivity to mitochondrial stress characterized by enhanced CHOP expression**<br>To test the contribution of HtrA2 to the transcriptional activation of a stress response, wild-type (WT) and HtrA2 KO primary mouse embryonic fibroblasts (MEFs) were challenged with an inducer of mitochondrial stress and their transcriptional response was monitored using microarray technology (experimental outline, Supplementary Figure S2a). WT and HtrA2 KO MEFs were chosen, as these cells have been shown earlier to display differential sensitivity to drug-induced stresses triggered by specific respiratory inhibitors such as rotenone, which specifically blocks complex I.[4] This analysis revealed that in HtrA2 KO MEFs, the number of rotenone-induced transcriptional changes was significantly enhanced, suggesting that loss of HtrA2 results in an enhanced transcriptional response (Figure 1a). Among the genes most highly induced by rotenone treatment in HtrA2 KO cells was the transcription factor CHOP (Supplementary Table S1), which is known to be induced in response to various stresses such as activation of the UPR^{ER}, UPR^{mt} and ISR.

Fraunhofer
SCAI

Field	raw_filename
Description	Raw file name for each sample, extracted from the raw file location
Examples	GSM318213.CEL.gz
GUIDELINE	This provides easy mapping of raw file names to the respective sample phenotype for gene expression analysis
SOURCE	GEO or ArrayExpress sample webpage

Field	cell_lines
Description	If the cell lines are used as samples or derived from the tissue extracetd
Examples	Embryonic mesenchymal cell line
GUIDELINE	Important for gene expression analysis and difference in gene expression in different cell lines
SOURCE	Image on Left (GEO): http://www.ncbi.nlm.nih.gov/geo/query/acc.cgi?acc=GSM26703<br>Image on Right (AE): https://www.ebi.ac.uk/arrayexpress/experiments/E-GEOD-36232/samples/<br><br>Cell line information can either be explicitly mentioned in AE/GEO or have to be searched from research paper linked to experiment. Cell line information is usually found in Materials and Methods section of paper, figure or supplementary files provided in research paper.

Field	additional_info
Description	Any interesting Data found by curator
Examples	
GUIDELINE	Some interesting outcome of experiment, if samples passed or failed QC, ethnicity, any outliers in data would not go unnoticed if they are noted in add_information column.
SOURCE	GEO/ AE files<br>Research paper

Field	id
Description	Internal identifier automatically generated by SQL database during upload
Examples	235
GUIDELINE	Important if we want to build relations between several tables

Field	annotation_remarks
Description	Sometimes used as the basis as to why the annotator annotated certain field
Examples	
GUIDELINE	This information is necessary if we want to trace back some ambiguous annotation as to why a certain field was annotated
SOURCE	GEO/ AE files<br>Research paper

Field	body_fluid
Description	If sample molecules are extracted from fluids of body
Examples	Whole Blood, Venous blood, peripheral blood
GUIDELINE	Separate analysis of gene expression changes, to differentiate it from the tissue expression
SOURCE	Body fluid information can either be explicitly mentioned in AE/GEO or have to be searched from research paper linked to experiment. Gender information is usually found in Materials and Methods section of paper, figure or supplementary files provided in research paper.

Field	brain_regions_or_tissue
Description	What region of brain or which tissue is used as sample
Examples	Brain Region: Caudate-Putamen, Prefrontal cortex, frontral cortex, neocortex, hippocampus, Whole Brain, ventral mid brain, striatum, cerebral cortex, left hippocampus, bilateral hippocampus, entorhinal cortex, embryonic brain, post natal brain
	Tissue: main olfactory epithelium, Granular Cell Layer
GUIDELINE	Vital for gene expression analysis based on brain region/ tissue bias
SOURCE	Image on Left (AE): https://www.ebi.ac.uk/arrayexpress/experiments/E-GEOD-31458/samples/<br>Image on Right (GEO): http://www.ncbi.nlm.nih.gov/pmc/articles/PMC2481328/<br><br>Brain and tissue type information can either be explicitly mentioned in AE/GEO or have to be searched from research paper linked to experiment. This information is usually found in Materials and Methods section of paper, figure or supplementary files provided in research paper. In one of the experiment exact information about brain region was found in figure caption (Image on right)

	Sample_source_name
	Pre-Frontal contex of naive AChE-S mice
	Pre-Frontal contex of naive AChE-S mice
	Pre-Frontal contex of naive FVB/N mice
	Pre-Frontal contex of naive FVB/N mice
	Pre-Frontal contex of naive AChE-R mice
	Pre-Frontal contex of naive AChE-R mice
ce	Pre-Frontal contex of MPTP-exposed FVB/N mice
ce	Pre-Frontal contex of MPTP-exposed FVB/N mice
nice	Pre-Frontal contex of MPTP-exposed AChE-R mice
nice	Pre-Frontal contex of MPTP-exposed AChE-R mice
nice	Pre-Frontal contex of MPTP-exposed AChE-S mice
nice	Pre-Frontal contex of MPTP-exposed AChE-S mice
	Caudate-Putamen of naive FVB/N mice
ce	Caudate-Putamen of naive FVB/N mice
ce	Caudate-Putamen of MPTP-exposed FVB/N mice
ce	Caudate-Putamen of MPTP-exposed FVB/N mice
	Caudate-Putamen of naive AChE-S mice
	Caudate-Putamen of naive AChE-S mice

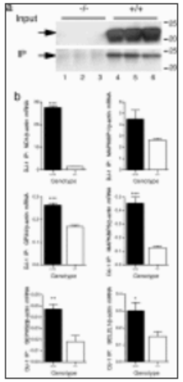

Fig. 2.

DJ-1 interacts with RNA *in vivo*. (*a*) IP for DJ-1 from whole brain lysates from knockout (lanes 1–3) or WT (lanes 4–6) mice. DJ-1 protein is absent from the knockout mice. (*b*) Validation by qRT-PCR after IP of DJ-1 from WT (closed bars) …

Fraunhofer
SCAI

Field	superseries
Description	If the current experiment is a superseries or not
Examples	Yes/ No (binomial value)
GUIDELINE	Gives information about superseries, this helps us to avoid duplicating the sample information
SOURCE	Image (GEO): http://www.ncbi.nlm.nih.gov/geo/query/acc.cgi?acc=GSE52024<br><br>Check in GEO experiment file if the sample is followed by the section of superseries. If superseries section is not found there, the current experiment is individual experiment and not a superseries.<br><br>This SuperSeries is composed of the following SubSeries:<br>GSE52022 Genome wide analysis of transcriptome and microRNAs in early stage of Alzheimer's disease (mRNA)<br>GSE52023 Genome wide analysis of transcriptome and microRNAs in early stage of Alzheimer's disease (microRNA)

Field	superseries_id
Description	Is this current experiment a part of superseries, then we need to map it to its superseries ID. If the experiment itself is the superseries or individual experiment then the column should be left empty
Examples	For GSE52022 experiment we include GSE52024 as superseries_id
GUIDELINE	Direct reference to the experiment superseries via a unique ID
SOURCE	Image on Left (GEO): http://www.ncbi.nlm.nih.gov/geo/query/acc.cgi?acc=GSE52024<br><br>Check in GEO experiment file if the samples are followed by the section of superseries information. If superseries section is not found there, the current experiment is individual experiment and has no superseries.<br><br>This SuperSeries is composed of the following SubSeries:<br>GSE52022 Genome wide analysis of transcriptome and microRNAs in early stage of Alzheimer's disease (mRNA)<br>GSE52023 Genome wide analysis of transcriptome and microRNAs in early stage of Alzheimer's disease (microRNA)<br>If this section is found, the series ID above would be superseries ID.<br>Series GSE52024 Query DataSets for GSE52024<br>Status: Public on Dec 01, 2013<br>Title: Genome wide analysis of transcriptome and microRNAs in early stage of Alzheimer's disease<br>Organism: Mus musculus<br>Experiment type: Expression profiling by array; Non-coding RNA profiling by high throughput sequencing<br>Summary: This SuperSeries is composed of the SubSeries listed below.<br>Overall design: Refer to individual Series

Field	disease_type
Description	Type of AD
Examples	sporadic AD, familial
GUIDELINE	Gives information to the cause of disease, if its genetically inherited or caused by other factors e.g. environment.
SOURCE	Image (Research Paper): http://hmg.oxfordjournals.org/content/19/20/3959.long<br>Image on Right (AE): https://www.ebi.ac.uk/arrayexpress/experiments/E-GEOD-36232/samples/<br><br>Disease type information can mainly be found from research paper of individual experiment.<br><br>Finally, we explored the hypothesis that loss of miRNA function could contribute to tau pathology in humans. To this end, we performed miRNA qRT-PCR from control and sporadic AD cases. Previous studies have shown that miR-16 is stably expressed in human tissues, including AD brain (25-27). Using this miRNA as internal control, we observed a significant decrease in miR-15a expression levels in AD brain when compared with healthy controls (Fig. 4C). There was no significant change in the expression of other miR-15 family members, including miR-15b and miR-195. We used as additional control the ubiquitously expressed let-7a, which is not affected in both groups. These results, together with previously published miRNA microarray data (27,28), strongly suggest that specific miR-15 family members, such as miR-15a, are affected in compromised brain displaying tau hyperphosphorylation.

Field	organism
Description	Sample organism
Examples	*Mus musculus*
GUIDELINE	To obtain information about the species from where the sample collected
SOURCE	Image on Top (GEO): http://www.ncbi.nlm.nih.gov/geo/query/acc.cgi?acc=GSE20447<br>Image on Bottom (AE): https://www.ebi.ac.uk/arrayexpress/experiments/E-GEOD-52024/<br><br>Organism information can be easily found in experiment GEO/ AE files.<br><br>Status Public on May 30, 2010<br>Title Neuronal microRNA response to Alzheimer's disease amyloid beta<br>Platform organisms Homo sapiens; Mus musculus; Rattus norvegicus<br>Sample organism Mus musculus<br>Experiment type Other<br><br>E-GEOD-52024 - Genome wide analysis of transcriptome and microRNAs in early stage of Alzheimer's disease<br>Status Released on 1 December 2013, last updated on 3 April 2014<br>Organism Mus musculus<br>Samples (16) Click for detailed sample information and links to data<br>└ found inside Mus musculus

Field	rat_weight
Description	Weight of rats
Examples	100-150g
GUIDELINE	To determine the metabolic changes
SOURCE	Rat weight information can either be explicitly mentioned in AE/GEO or have to be searched from research paper linked to experiment. Gender information is usually found in Materials and Methods section of paper, figure or supplementary files provided in research paper.

Field	platform_id
Description	ID of the platform used for the experiment
Examples	GPL339, GPL16354
GUIDELINE	Quick reference to the platform information of the platform used. Supports in probe annotation information
SOURCE	Image (GEO): http://www.ncbi.nlm.nih.gov/geo/query/acc.cgi?acc=GSE47036<br>Image on bottom (AE): https://www.ebi.ac.uk/arrayexpress/experiments/E-GEOD-13691/<br><br>Platform ID information can be found in GEO experiment file. There can be different platforms used for different sample. Below screenshot shows information of 2 platforms for a single experiment. Each sample is mapped to the respective platforms.

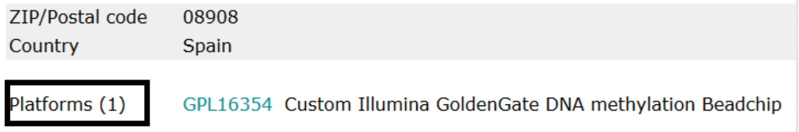

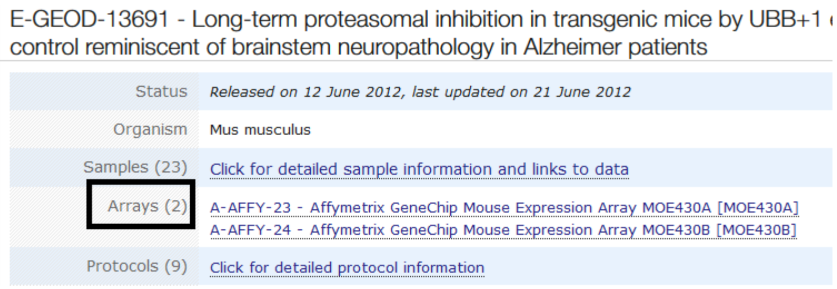

Field	type_of_treatment (Animal models only)
Description	Information about the type of treatment used on mice/rat
Examples	MPTP treated, untreated (if the sample is not treated), treated with Kainic acid, treated with saline or vehicle, rasagiline, given chronically post-MPTP, water treated (control), PBS mixed with 18 mix of ethanol (Sigma-Aldrich, St Louis, MO, USA) and neobee® M-5 oil (Stepan Company, Northfield, IL, USA) as vehicle.
GUIDELINE	Assists in identification of correlation between type of treatment used to induce disease in mice/rat, or treatment of induced disease
SOURCE	Image (GEO): http://www.ncbi.nlm.nih.gov/geo/query/acc.cgi?acc=GSM862523<br><br>Information about type of treatment is mostly found in materials and methods section of paper. However, entire paper should be thoroughly read especially the supplementary files to find exact treatment type information. Sometimes, it can also be found from GEO (in treatment protocol section of sample file in GEO) and AE experiment files.<br><br>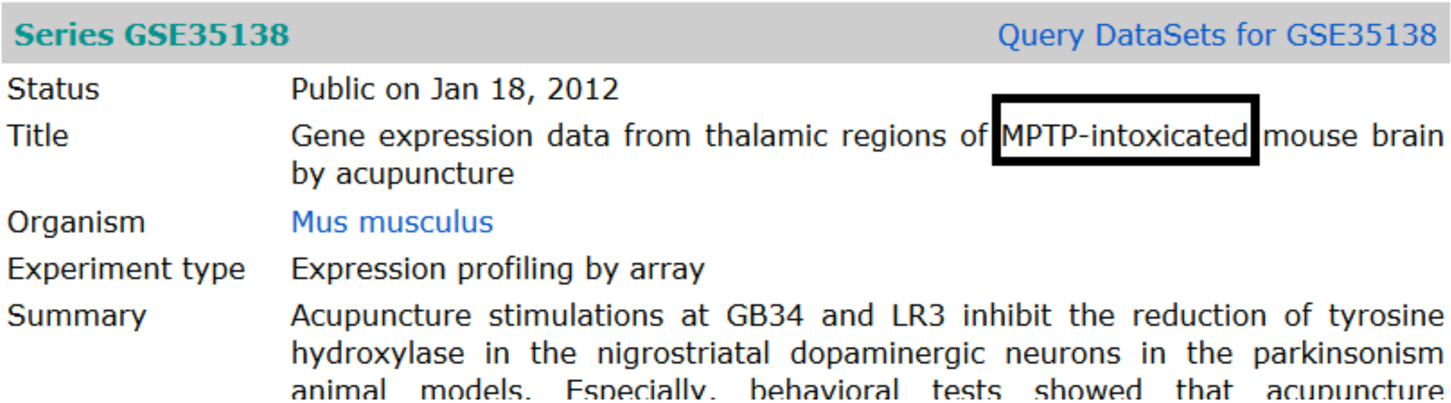<br>Series GSE35138 Query DataSets for GSE35138<br>Status Public on Jan 18, 2012<br>Title Gene expression data from thalamic regions of MPTP-intoxicated mouse brain by acupuncture<br>Organism Mus musculus<br>Experiment type Expression profiling by array<br>Summary Acupuncture stimulations at GB34 and LR3 inhibit the reduction of tyrosine hydroxylase in the nigrostriatal dopaminergic neurons in the parkinsonism animal models. Especially, behavioral tests showed that acupuncture

Field	physical_injury (Animal models only)
Description	Information about the physical injury done on mice/rat (either for generation of mice/rat model or to assess injury as treatment
Examples	acupoints acupuncture-treated, ischemia/reperfusion (I/R) injury 2h, Sham control, nonacupoints acupuncture-treated
GUIDELINE	Identification of the process used to induce disease in mice/rat through injury
SOURCE	Image on Left (GEO): http://www.ncbi.nlm.nih.gov/geo/query/acc.cgi?acc=GSE35138<br>Image on Right (AE): https://www.ebi.ac.uk/arrayexpress/experiments/E-GEOD-23162/samples/<br><br>Information about type of physical injury is mostly found in materials and methods section of paper. However, entire paper should be thoroughly read especially the supplementary files to find exact injury method information. Sometimes, it can also be found from GEO (in treatment protocol section of sample file in GEO) and AE experiment files.

2h	Gpx1-/-	ischemia/reperfusion (I/R) injury
2h	Gpx1-/-	ischemia/reperfusion (I/R) injury
2h	Gpx1-/-	ischemia/reperfusion (I/R) injury
2h	Gpx1-/-	ischemia/reperfusion (I/R) injury
8h	Gpx1-/-	ischemia/reperfusion (I/R) injury
8h	Gpx1-/-	ischemia/reperfusion (I/R) injury
8h	Gpx1-/-	ischemia/reperfusion (I/R) injury
8h	Gpx1-/-	ischemia/reperfusion (I/R) injury
24h	Gpx1-/-	ischemia/reperfusion (I/R) injury
24h	Gpx1-/-	ischemia/reperfusion (I/R) injury
24h	Gpx1-/-	ischemia/reperfusion (I/R) injury
24h	Gpx1-/-	ischemia/reperfusion (I/R) injury
Ctrl	Gpx1-/-	sham

Field	dosage (Animal models only)
Description	Information about the amount of treatment, at what interval and for what interval done on mice/rat
Examples	MPTP treated: intraperitoneally injected with saline 0.9% (100 µl) once daily for four weeks<br>Saline treated: 15mg/kg in 0.2 ml volume Saline treated_control<br>Water: recieved Water for 14 days orally
GUIDELINE	Relevant to obtain the amout of certain substance used to induce or treat the disease in animal models
SOURCE	Image on Left (GEO): http://www.ncbi.nlm.nih.gov/geo/query/acc.cgi?acc=GSM26703<br>Image on Right (AE): https://www.ebi.ac.uk/arrayexpress/experiments/E-GEOD-36232/samples/<br><br>Information about dosage is mostly found in materials and methods section of paper. However, entire paper should be thoroughly read especially the supplementary files to find exact dosage information. Sometimes, it can also be found from GEO (in treatment protocol section of sample file in GEO) and AE experiment files.<br><br>Treatment protocol: While mice in the control group (n=9) were intraperitoneally injected with saline 0.9% (100 µl) once daily for four weeks, the mice in the MPTP group (n=9) were intraperitoneally injected with MPTP-HCl (20 mg/kg of free base) dissolved in saline 0.9% (100 µl) at twenty-four hr intervals for four weeks to produce the sustained chronic model of parkinsonism. Acupuncture was performed by hand two hr after the first MPTP injection and then at forty-eight hr intervals (fourteen total sessions). The acupuncture procedure (acupoint GB34 (Yanglingquan) and acupoint LR3 (Taichong) for acupoints; both sides of the hips for non-acupoints) was performed as previously reported. Mice in the acupoints group were immobilized by hand two hr after MPTP administration. Acupuncture needles were inserted bilaterally to depths of 1 mm at acupoint LR3 and 3 mm at acupoint GB34, and then turned at a rate of two spins per sec for fifteen sec as reported in a previous study. For the non-acupoints group, the needles were inserted to depths of 3 mm at both sides of the hips rather than at the acupoints GB34 and LR3 for the acupoints group, and then the same procedures were performed as with the acupoints.

Field	functional_effect
Description	Functional effects observed in microarray, sequencing or other experiment as a result of physical injury or chemical treatment
Examples	mouse affected by MPTP and acupuncture only at the acupoints, are responsible for exerting in the striatal regions the inhibitory effect of acupuncture at the acupoints on MPTP-induced striatal degeneration.**:** Acupuncture at acupoints GB34 and LR3 has been reported to inhibit nigrostriatal degeneration in parkinsonism models, yet the genes related to this preventive effect of acupuncture on the nigrostriatal dopaminergic system remain elusive. SMARCA4 gene was downregulated in mTLE patients.
GUIDELINE	Correlation between treatment and gene expression changes seen in experiment by the authors
SOURCE	Functional effects is a free text information field where different functional effects observed in sample before and after treatment observed is included.<br><br>Functional effect can be found from result, discussion, conclusion or other analytical sections of research paper.<br><br>Different functional effect are separated by a ":" (as seen in example above)<br><br>Functional effects field can also include external links or supplementary files if information about gene expression is large.

Fraunhofer
SCAI

Field	behaviour
Description	Physical traits seen in mice/rat before and/or after treatment. Behavioural changes that can be notified in diseased patient during the experiment
Examples	behavioral traits in the mice/rat are akin to PD patients, deficits in fear conditioning, central breathing dysfunction, altered breathing patterns, high respiratory rate, increased duration of inspiration, results of Active Avoidance Learning test, results of novel object recognition test, results of the accelerating rotarod test, results of the grip strength test, results of the test of hanging from an inverted cage lid, overall activity in the novel open field arena, Y maze test, PA(Passive avoidance paradigm) assessment, put NA if not available. Tremor (shaking of hands)
GUIDELINE	Gives correlation between behaviour change and gene expression changes.
SOURCE	Image (Reference paper): http://www.ncbi.nlm.nih.gov/pmc/articles/PMC3319057/<br><br>Behaviour information would rarely be explicitly mentioned in AE/GEO so it has to be searched from research paper linked to experiment. Behaviour information is usually found supplementary tables provided with the research paper. It would be difficult to find behaviour information and demands reading research paper thoroughly.<br><br>two different toxicological mouse models of PD created using 1-methyl-4-phenyl-1,2,3,6-tetrahydropyridine (MPTP) and methamphetamine (METH). MPTP has been shown to induce Parkinsonism in mice with recapitulation of the hallmark cellular pathology, death of dopaminergic neurons in the SN, which decreases DA input to the target organ, the striatum.[12,13] MPTP probably acts through the inhibition of oxidative phosphorylation in dopaminergic neurons. [14] METH-treated mice also exhibit cell death of dopaminergic neurons in the SN, and although the parallels to the human disease are less strong, the behavioral traits in the mice are akin to PD patients.[15,16] METH stimulates catecholamine release, although the mechanisms by which large doses result in a PD model are unclear.[17,18]

Fraunhofer
SCAI

<table>
<tr><th>Field</th><th>mouse_name or rat_name</th></tr>
<tr><td>Description</td><td>Vendor/ commercial name of mouse used</td></tr>
<tr><td>Examples</td><td>C57/Bl6, C57BL/6-129, crossed Tg2576 APP transgenic mice/rat with PS1 M146V homozygous (PS1M146V/M146V) mice/rat: Alzheimers Disease, B6/SJL, B6/SJL (for hemizygous 5xFAD mice/rat) and C57BL/6J (for eIF2α+/S51A mice/rat), FVB/N mice/rat, Sprague-Dawley rat. Put NA if not availaable</td></tr>
<tr><td>GUIDELINE</td><td>Relates the mouse used with gene expression changes. This could support in understanding the effects specific to a mouse model</td></tr>
<tr><td>SOURCE</td><td>Image on Left (AE): https://www.ebi.ac.uk/arrayexpress/experiments/E-GEOD-47036/samples/
Image on Right (GEO): http://www.ncbi.nlm.nih.gov/geo/query/acc.cgi?acc=GSM1165975

Information about mouse is mostly found in materials and methods section of paper. However, entire paper should be thoroughly read especially the supplementary files to find exact mouse nformation. Sometimes, it can also be found from GEO (in growth protocol section of sample file in GEO) and AE experiment files. Mouse breeding information is also included.
<table>
<tr><th>genetic background</th><th>genotype</th></tr>
<tr><td>C57BL/6-129</td><td>3TG</td></tr>
<tr><td>C57BL/6-129</td><td>3TG</td></tr>
<tr><td>C57BL/6-129</td><td>3TG</td></tr>
<tr><td>C57BL/6-129</td><td>3TG</td></tr>
<tr><td>C57BL/6-129</td><td>APP/PSEN1</td></tr>
<tr><td>C57BL/6-129</td><td>APP/PSEN1</td></tr>
<tr><td>C57BL/6-129</td><td>APP/PSEN1</td></tr>
<tr><td>C57BL/6-129</td><td>APP/PSEN1</td></tr>
<tr><td>C57BL/6-129</td><td>APP/PSEN1</td></tr>
<tr><td>C57BL/6</td><td>WT</td></tr>
<tr><td>C57BL/6</td><td>WT</td></tr>
<tr><td>C57BL/6</td><td>WT</td></tr>
<tr><td>C57BL/6</td><td>WT</td></tr>
<tr><td>C57BL/6</td><td>WT</td></tr>
</table>
<table>
<tr><td>Status</td><td>Public on Sep 19, 2013</td></tr>
<tr><td>Title</td><td>APP/PS1M146V/+</td></tr>
<tr><td>Sample type</td><td>RNA</td></tr>
<tr><td>Source name</td><td>APP/PS1M146V/+</td></tr>
<tr><td>Organism</td><td>Mus musculus</td></tr>
<tr><td>Characteristics</td><td>disease state: alzheimer's disease
tissue: bilateral hippocampus
genotype/variation: APP/PS1M146V/+</td></tr>
<tr><td>Treatment protocol</td><td>No treatment</td></tr>
<tr><td>Growth protocol</td><td>APP/PS1M146V/+ mice were generated by crossed Tg2576 APP transgenic mice with PS1 M146V homozygous (PS1M146V/M146V) mice. All mice were feed and housed according to standard procedures.</td></tr>
<tr><td>Extracted molecule</td><td>total RNA</td></tr>
</table></td></tr>
</table>
