# Supplementary material for: NeuroTransDB: highly curated and structured transcriptomic metadata for neurodegenerative diseases
Source: Database (Oxford). 2015 Oct 15;2015:bav099. doi: 10.1093/database/bav099 (PMC4608514; doi:10.1093/database/bav099)
Supplement: Supplementary Data [file supp_bav099_supplementary_data.zip › SupplementaryTableS1.doc]

| **Experiment ID** | **Contact Reason** | **Response of the authors** |
| --- | --- | --- |
| **GSE47036** | Mismatch in sample labelling between GEO database and the associated publication | Provided us the correct mapping file, in addition authors corrected the error in GEO database |
| **GSE47038** | Difference in number of samples between GEO (58 samples) and ArrayExpress (64 samples) | ArrayExpress synced the sample count to GEO database |
| **GSE29652** | Difficulty in mapping the sample information from GEO to the detailed information provided in publication (Table 1) | The authors provided us additional file mapped to Source name (*cf.* GEO sample page) along with detailed information of how to map these different IDs to the case information in Table 1 |
| **GSE36980** | Could not map the patient information (Subject No.) provided in Supplementary Table 2 to the GEO sample IDs | Authors provided additional supplementary file which contained GEO sample IDs mapped to subject numbers in Supplementary Table S2 |
| **GSE26927** | Unable to map the Cohort characteristics provide in Table 1 (or Supplementary Online Resource 1) of the paper with sample information in GEO. In addition, the number of samples used in the analysis (113), as described in the paper, differs from the number of samples in GEO (118). | Author provided the mapping information of sample title with cohort characteristics. Additionally, the authors reported that only 113 samples out of 118 were of good quality, thus the difference in sample numbers. |

**Table S1**: **Detailed listing of GEO experiment IDs where we contacted the authors for missing or mismatched information.** Along with the experiment IDs we provide the reason for contacting the authors and their response.
