# Supplementary material for: NeuroTransDB: highly curated and structured transcriptomic metadata for neurodegenerative diseases
Source: Database (Oxford). 2015 Oct 15;2015:bav099. doi: 10.1093/database/bav099 (PMC4608514; doi:10.1093/database/bav099)
Supplement: Supplementary Data [file supp_bav099_supplementary_data.zip › SupplementaryTableS2.doc]

| **Sample ID from GEO** | **Sample title from GEO (resemble patient ID)** | **Associated sample age provided in GEO website** | **Patient ID in supplementary file** | **Associated gender information in supplementary file** |
| --- | --- | --- | --- | --- |
| **GSM466881** | PD rep1 | Male | PD8 | M |
| **GSM466882** | PD rep2 | Female | PD9 | M |
| **GSM466883** | PD rep3 | Male | PD11 | M |
| **GSM466884** | PD rep4 | Male | PD13 | M |
| **GSM466885** | PD rep5 | Male | PD14 | M |
| **GSM466886** | PD rep6 | Male | PD15 | M |
| **GSM466887** | PD rep7 | Male | PD15x | M |
| **GSM466888** | PD rep8 | Female | PD17 | F |
| **GSM466889** | PD rep9 | Male | PD18 | M |
| **GSM466890** | PD rep11 | Female | PD20 | F |
| **GSM466891** | PD rep12 | Male | PD21 | M |
| **GSM466892** | PD rep13 | Male | PD22 | M |
| **GSM466893** | PD rep14 | Male | PD22x | M |
| **GSM466894** | PD rep15 | Male | PD23 | M |
| **GSM466895** | PD rep16 | Male | PD23x | M |
| **GSM466896** | PD rep17 | Female | PD27 | M |
| **GSM466897** | PD rep18 | Male | PD30 | M |
| **GSM466898** | Ctrl rep_2 | Female | CNT4 | F |
| **GSM466899** | Ctrl rep_3 | Female | CNT5 | F |
| **GSM466900** | Ctrl rep_4 | Male | CNT6 | F |
| **GSM466901** | Ctrl rep_5 | Male | CNT15 | F |
| **GSM466902** | Ctrl rep_6 | Male | CNT16 | F |
| **GSM466903** | Ctrl rep_7 | Female | CNT18 | F |
| **GSM466904** | Ctrl rep_8 | Female | CNT19 | M |
| **GSM466905** | Ctrl rep_9 | Male | CNT20 | M |
| **GSM466906** | Ctrl rep_10 | Male | CNT21 | F |
| **GSM466907** | Ctrl rep_11 | Female | CNT24 | M |
| **GSM466908** | Ctrl rep_12 | Male | CNT 25 | F |

**Table S2**: **Detailed mapping of GEO’s Sample ID and Patient ID (from supplementary Table S6 of the associated publication) along with their associated gender information of experiment GSE18838.** Here we mapped the Sample and Patient IDs with an assumption that they are in natural sorted order. Samples for which we could not map the meta-data information between GEO archive and supplementary file (Table S6) of the associated published article are highlighted in green. The difference in male/female ratio between GEO samples (male/female: 19/9) and Supplementary Table S6 (male/female: 18/8) provides enough evidence that the information is misleading.
